# Marked changes in dendritic structure and spine density precede significant neuronal death in vulnerable cortical pyramidal neuron populations in the SOD1^G93A^ mouse model of amyotrophic lateral sclerosis

**DOI:** 10.1186/s40478-016-0347-y

**Published:** 2016-08-04

**Authors:** Matthew J. Fogarty, Erica W. H. Mu, Peter G. Noakes, Nickolas A. Lavidis, Mark C. Bellingham

**Affiliations:** 1 School of Biomedical Sciences, The University of Queensland, St Lucia, Australia; 2 Queensland Brain Institute, The University of Queensland, St Lucia, Australia

**Keywords:** Dendrite, Spine density, Cortex

## Abstract

Amyotrophic lateral sclerosis (ALS) is characterised by the death of upper (corticospinal) and lower motor neurons (MNs) with progressive muscle weakness. This incurable disease is clinically heterogeneous and its aetiology remains unknown. Increased excitability of corticospinal MNs has been observed prior to symptoms in human and rodent studies. Increased excitability has been correlated with structural changes in neuronal dendritic arbors and spines for decades. Here, using a modified Golgi-Cox staining method, we have made the first longitudinal study examining the dendrites of pyramidal neurons from the motor cortex, medial pre-frontal cortex, somatosensory cortex and entorhinal cortex of hSOD1^G93A^ (SOD1) mice compared to wild-type (WT) littermate controls at postnatal (P) days 8–15, 28–35, 65–75 and 120. Progressive decreases in dendritic length and spine density commencing at pre-symptomatic ages (P8-15 or P28-35) were observed in layer V pyramidal neurons within the motor cortex and medial pre-frontal cortex of SOD1 mice compared to WT mice. Spine loss without concurrent dendritic pathology was present in the pyramidal neurons of the somatosensory cortex from disease-onset (P65-75). Our results from the SOD1 model suggest that dendritic and dendritic spine changes foreshadow and underpin the neuromotor phenotypes present in ALS and may contribute to the varied cognitive, executive function and extra-motor symptoms commonly seen in ALS patients. Determining if these phenomena are compensatory or maladaptive may help explain differential susceptibility of neurons to degeneration in ALS.

## Introduction

Amyotrophic lateral sclerosis (ALS) is the most common motor neuron disease [11] and is clinically characterised by the death of upper and lower motor neurons (MNs) and the degeneration of the corticospinal tract [10, 25]. Loss of MNs leads inexorably to muscle wastage and weakness, progressing to eventual death within 3–5 years from diagnosis [25], typically from respiratory complication or failure [12]. *Post-mortem* evaluations of both upper and lower MNs using the Golgi-Cox impregnation technique have shown structural defects, including dendritic retraction and spine loss [24, 27, 32, 65]. Imaging studies have shown thinning of the regions containing upper MNs as well as their axonal tract projections in ALS patients [55, 71, 74]. Taken together, these structural abnormalities correlate with measurements of cortical hyper-excitability [16, 41, 64, 72, 77] before diagnosis in certain ALS patients [73], suggesting that structure/function alterations in neurons of these regions occurs during a protracted preclinical phase, playing a key role in disease pathogenesis [15, 68].

The stream of excitatory and inhibitory synaptic inputs are integrated by the dendritic structure of the neuron, determining whether the neuron generates an action potential [39]. Changes in the type or frequency of neurotransmitter signalling induces alterations in dendritic length and dendritic spine density during normal development and aging, in addition to pathologic alterations in psychiatric and neurodegenerative diseases [39, 54]. In ALS, these changes are linked to one of the major proposed aetiological mechanisms for the disease, glutamate-induced excitotoxicity [7, 8, 14, 16, 20, 41, 53, 63, 68, 69, 77].

Structural abnormalities in the dendritic arbors and/or dendritic spines of neurons from the most widely used ALS rodent model, the hSOD1^G93A^ transgenic mouse (SOD1), have been reported in upper MNs from the motor cortex [20, 30, 53, 58] and medial pre-frontal cortex (MPFC) [57], as well as lower MNs in the brainstem [69] and spinal cord [40]. Although these studies provide some insight into individual components of the neuro-motor network, there remains a need to characterize structural abnormalities in motor, cognitive, sensory and extra-motor regions at differing stages of disease, to reveal whether changes in neuron structure occur prior to, and are confined to, vulnerable neurons, compared to non-vulnerable neurons. Here, we have characterized changes in neuronal structure in motor-related populations (motor cortex and somatosensory cortex) severely affected in ALS [10, 11, 15, 24, 25, 27, 32, 63, 65], and in cortical regions associated with cognitive function deficits (the MPFC and entorhinal cortex) in a subset of ALS patients [37, 38, 47, 63].

Our major findings are the demonstration of early and progressive dendritic degeneration and spine loss in upper MNs of the motor cortex, while pyramidal neurons of the MPFC showed early basal arbor growth, followed by basal and apical arbor retraction and spine loss. Unexpectedly, changes were seen in layer II/III pyramidal neurons of the motor cortex, showing early loss of apical spines and later dendritic arbor retraction, and layer V of the somatosensory cortex, showing apical spine loss at symptom onset. No significant changes were seen in pyramidal neurons of the entorhinal cortex or layer II/III of the somatosensory cortex. Changes in motor and somatosensory cortices and MPFC occurred concomitant with decreased cortical thickness.

## Materials and methods

### Ethics statement

A total of 52 age- and litter-matched wild-type (WT) and heterozygous transgenic mice overexpressing the hSOD1^G93A^ mutation (SOD1) were used. All procedures were approved by The University of Queensland Animal Ethics Committee and were conducted in accordance with the Queensland Government Animal Research Act 2001, associated Animal Care and Protection Regulations (2002 and 2008), as well as the Australian Code for the Care and Use of Animals for Scientific Purposes, 8th Edition (National Health and Medical Research Council, 2013).

### Golgi-Cox impregnation and processing

Age and litter-matched mice from postnatal (P) days P8-15, P28-25, P65-75 and P120 (±10 days), corresponding to previously characterized early neonatal (intrinsic and synaptic neuronal hyper-excitability) [20, 69], pre-symptomatic (no overt symptoms, synaptic and structural neuronal deficits) [20, 45, 53, 57], disease onset (early MN loss and muscle weakness) [30, 35, 45, 60] and mid-disease stages (marked muscle mass and agility loss) respectively [35]. Mice were anaesthetized with an intra-peritoneal injection of sodium pentobarbitone (60–80 mg/kg, Vetcare) and exsanguinated intracardially using a heparinized needle (Sigma-Aldrich). Whole brain tissues were then incubated for 5 days at 37 °C in the dark in a modified rapid Golgi-Cox solution that contained 5 % potassium dichromate, 5 % potassium chromate, 5 % mercuric chloride, as described previously [34, 52] (all chemicals Sigma-Aldrich).

Following incubation in Golgi-Cox solution, 300 μm thick off-coronal (15° rostral rotation) cortical slices (embedded in 10 % agarose block in 0.1 M phosphate buffered saline) were cut using a vibrating Zeiss Hyrax V50 microtome (Carl Zeiss). Cut slices were sequentially placed in 24-well plates filled with 30 % sucrose in 0.1 M phosphate buffered saline for 30 min, before being dehydrated in 50 % ethanol for 5 min and then incubated in 0.1 M ammonium hydroxide solution for 30 min. Slices were rinsed in distilled water twice for 5 min, before being incubated in aluminium sulphate-based Fujihunt photo fixer (Fuijfilm, Singapore) for 30 min while protected from light exposure. Slices were again rinsed in distilled water twice for 5 min, before dehydrating in ascending concentrations of ethanol (70 %, 90 %, 95 %, and three rinses of 100 %) for 5 min each. Slices were transferred to a chloroform: xylene: alcohol (CXA) solution (1:1:1 ratio) for 10 min. From CXA solution, slices were cleared in two changes of xylene for 5 min each and mounted using DPX (Sigma-Aldrich) on Superfrost Plus (Lomb Menzel Glaser) slides. Slides were air dried and stored in the dark. We note that aged mice required longer xylene (extra 5 min) clearing due to a higher fatty content of the neurological tissues.

### Cortical region selection and morphometry

**Fig. 1:**
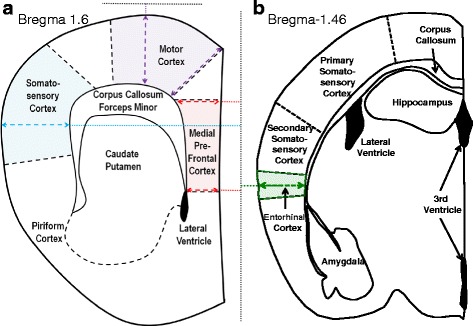
Cortical regions used for measuring thickness and for tracing neurons. Lines parallel or perpendicular to the axis of the midline (*dotted lines*) were used to measure: **a** the thickness of the motor cortex (mean of purple dashed arrows from the corpus callosum to the pia), the MPFC (means of the red dashed arrows from the corpus callosum to the midline), the somatosensory cortex (the length of the blue arrow from the lateral fork of the corpus callosum to the most lateral edge of the pia) and (**b**) the entorhinal cortex (the length of green arrow from the lateral border of the corpus callosum to the most lateral edge of the pia). **a** and **b** show the motor cortex (*purple*), MPFC (*red*), somatosensory cortex (*blue*) and entorhinal cortex (*green*) regions to be used to select neurons for dendritic morphology assessment were defined using the aid of a mouse brain atlas [21]

Off-coronal sections between bregma ~1.9 and 0.6 mm were used to quantify cortex thickness and to select neurons to trace dendritic arbors in the motor cortex, somatosensory cortex and MPFC (Fig. 1). Off-coronal sections between bregma ~ −1.1 and −2.6 mm were used to select neurons to trace dendritic arbors in the entorhinal cortex. Regional boundaries were defined with the aid of a mouse brain atlas [21]. Motor cortical thickness for a particular brain slice was assessed by using the mean of two line measurements, with one line measuring from the dorso medial edge of the corpus callosum through the furthest dorso-medial extent of the cortical pia (Fig. 1). The second line measured (perpendicular to the horizontal axis) from the dorsal edge of the corpus callosum to the furthest dorsal extent of the cortical pia (Fig. 1).

Somatosensory cortical thickness for an individual brain slice was measured using a line from the lateral edge of the corpus callosum to the most lateral extent of the cortical pia (Fig. 1).

The entorhinal cortical thickness was measured using a line perpendicular to the vertical axis from the white matter to the pia (Fig. 1).

Medial pre-frontal cortical thickness for an individual brain slice was quantified by using the mean of two line measurements, with one line measuring (perpendicular to the vertical axis) from the medial edge of the corpus callosum through the furthest medial extent of the cortical pia at the dorsal border of the MPFC (Fig. 1). The second line was measured in an identical fashion at the ventral border of the MPFC (Fig. 1).

### Neuronal tracing

Morphological properties (dendritic branching, length and dendritic spines) of Golgi-impregnated neurons were traced under the 63x objective (NA 1.4) of an Axoskop 2 microscope (Carl Zeiss) with an automated *z*-stage controlled by Neurolucida™ software (MBF Bioscience Inc.) in a manner identical to previous reports, with small processes classified as spines only if they were not >3 μm in length and not >0.8 μm in cross-sectional diameter [20, 26]. To be included in the data set, a pyramidal neuron had to have a minimum of 3 intact basal dendritic trees and an apical dendrite that did not exit the plane of the off-coronal brain slice before reaching the dendritic terminus. Apical dendrites included the trunk emanating from the soma and all of the emanating accessory branches and tufts related to the tree as per previous studies [20, 33]. Dendritic spines were assessed along the entirety of the apical or dendritic arbor, and are an estimate identical to that in previous studies [20]. The following quantitative data was generated from these tracings using NeuroExplorer™ software (MBF Bioscience); i) soma (irregular ellipsoid) volume (based on cross-sectional area slices in a manner identical to previous studies [31]), ii) total arbor length (the entirety of the dendritic arbor length summed), iii) apical or basal length (the length of single apical tree or the length of all of the basal trees summed) iv) mean basal tree or mean dendritic length (the mean length of each basal or dendritic tree emanating from the soma), v) apical and basal reach (the radial distance the farthest apical or basal dendrite ending is from the neuronal soma), vi) apical and basal ramifications (the number of times a dendrite bifurcates before terminating, a measure of neuronal complexity) and vii) apical or basal dendritic spine density (the number of dendritic spines per 100 μm of dendrite). A total length of 2.15 m of dendrite was traced in the 724 neurons that were used in this study.

### Imaging

For figures presenting neuronal dendritic arbors, minimum intensity projections of *z*-stacks (with a *z*-step size of 2 μm between images and a stack depth ranging between 20–100 images) were combined to form mosaics of entire cells. The extent of the overlaid tracing file was used to determine the area to image. The ‘tessellated’ appearance of the mosaics is due to differences in the thickness of the individual *z*-stacks. For images and figures presenting dendritic spines, a single 100x objective (Carl Zeiss) image at a single focal plane was used to create representative examples of spine density.

### Statistical methods

All analysis was done with Prism 6 (Graphpad). Data are expressed as mean ± SEM. For all the data reported in figures, tables and text, the *n* is the number of mice in that age cohort. The mean ± SEM calculated from all of the sampled neurons in that brain region for that particular animal, as for other Golgi-Cox impregnation studies [34], with the number of neurons a particular value is derived from reported in the tables. To assess the data for normality, the cardinal properties of all sampled neurons (total dendritic arbor, apical spine density and basal dendritic spine density) for a particular age/genotype were assessed using a D’Agostino and Pearson omnibus test for normality; the *P* value for this test ranged between 0.09 and 0.98, suggesting that ANOVA tests were appropriate. Statistically significant differences were determined using two-way ANOVAs with Bonferroni’s post-tests applied to data sets that showed significant differences with regard to genotype, where adjusted *P* (adj. *P*) values were shown by *P < 0.05, **P < 0.01, ***P < 0.001 and ****P < 0.0001, and were considered statistically significant. Percentage changes are reported in relation to the WT mean at significantly different age groups. The researcher who performed tracings (MJF) was blind to the animal genotype.

## Results

### Cortical thickness is reduced in SOD1 mice by P28-35 in the motor cortex, and by P120 in the MPFC and in the somatosensory cortex, compared to WT controls

Imaging studies have shown thinning of various cortical regions, including those containing upper MNs, as well as their axonal tract projections in ALS patients [55, 71, 74]. Although a variety of pathogenic process may be contributing to cortical thinning in ALS (neuronal death, dendritic degeneration and projection axon loss), we assessed whether this occurred in the SOD1 mice at different ages and brain regions, namely the motor cortex, the MPFC, entorhinal cortex and the somatosensory cortex.

Motor cortex thickness was reduced by 20 % in P28-30 SOD1 mice, compared to WT controls (*P* = 0.012; Table 1). By P65-75, motor cortex thickness of SOD1 mice was further reduced, by 26 %, compared to WT controls (*P* = 0.0016; Table 1). By P120, the oldest age studied, motor cortex thickness in SOD1 mice was reduced by 24 %, compared to WT controls (*P* = 0.0005; Table 1).

**Table 1:** Cortical thickness for the motor cortex, MPFC, somatosensory cortex and entorhinal cortex. All data presented as mean ± SEM

Region	P8-15 (*n*) Neonatal	P28-35 (*n*) Pre-symptomatic	P65-75 (*n*) Onset	P120 (*n*) Mid-disease
Motor cortex	WT: 1057 ± 23.8 (*7*)	WT: 1494 ± 48.9 (*5*)	WT: 1440 ± 72.8 (*5*)	WT: 1487 ± 69.9 (*8*)
	SOD1: 1095 ± 38.2 (*5*)	SOD1: 1189 ± 112.9 (*5*)	SOD1: 1069 ± 62.5 (*5*)	SOD1: 1136 ± 40.4 (*12*)
	Adj. *P* > 0.99	Adj. *P* = 0.012*	Adj. *P* = 0.002**	Adj. *P* = 0.0005***
MPFC	WT: 489 ± 38.8 (*7*)	WT: 645 ± 25.6 (*5*)	WT: 613 ± 36.2 (*5*)	WT: 697 ± 39.6 (*8*)
	SOD1: 495 ± 11.4 (*5*)	SOD1: 602 ± 53.7 (*5*)	SOD1: 541 ± 38.3 (*5*)	SOD1: 507 ± 21.2 (*12*)
	Adj. *P* > 0.99	Adj. *P* > 0.99	Adj. *P* > 0.99	Adj. *P* < 0.0001****
Somatosensory cortex	WT: 995 ± 46.0 (*7*)	WT: 1259 ± 66.6 (*5*)	WT: 1223 ± 22.1 (*5*)	WT: 1211 ± 39.8 (*8*)
	SOD1: 1002 ± 22.3 (*5*)	SOD1: 1148 ± 26.0 (*5*)	SOD1: 1242 ± 40.2 (*5*)	SOD1: 997 ± 39.8 (*12*)
	Adj. *P* > 0.99	Adj. *P* = 0.49	Adj. *P* > 0.99	Adj. *P* = 0.0005***
Entorhinal cortex	WT: 733 ± 38.1 (*7*)	WT: 960 ± 30.1 (*5*)	WT: 907 ± 28.7 (*5*)	WT: 956 ± 42.7 (*8*)
	SOD1: 785 ± 55.6 (*5*)	SOD1: 983 ± 78.6 (*5*)	SOD1: 904 ± 36.3 (*5*)	SOD1: 833 ± 57.8 (*12*)
	Not Significant	Not Significant	Not Significant	Not Significant

All analyses were unpaired two-way ANOVAs with significant differences in the motor cortex (Age: *P* = 0.0005***; Genotype: *P* < 0.0001****), MPFC (Age: *P* = 0.0053**; Genotype: *P* = 0.0054**), somatosensory cortex (Age: *P* < 0.0001****; Genotype: *P* = 0.0251*) and entorhinal cortex (Age: *P* < 0.0072**; Genotype: *P* = 0.7548). Bonferroni post-tests were used to compare the effect of genotype at each age group, with the adjusted *P*-value (adj. *P*) reported in the table for regions that had significant genotype effects

For the MPFC and for the somatosensory cortex, we observed no significant difference in cortical thickness at P8-15, P28-35 or P65-75 in SOD1 mice, compared to WT controls (Table 1). By P120, SOD1 mice had reduced MPFC thickness of 27 %, compared to WT controls (adj. *P* < 0.0001; Table 1), and somatosensory cortex thickness was reduced by 18 % in SOD1 mice, compared to WT controls (adj. *P* < 0.0005; Table 1). There was no difference in the entorhinal cortical thickness between control and SOD1 mice at any age (*P* = 0.75).

Taken together, these results indicate that degenerative processes in the motor cortex precede those of the MPFC and somatosensory cortex, and changes in cortical thickness are evident before neuronal loss in layer V of the motor cortex is significant, at P60 [30, 45]. To ascertain whether changes in cortical thickness were correlated with structural changes in pyramidal neurons of these cortical areas, we next characterised the dendritic arbors and dendritic spine densities of pyramidal neurons in layer V (LVPNs) and layer II/III (LII-IIIPNs) from the motor cortex and somatosensory cortex, layer II/III from the entorhinal cortex and layer V pyramidal neurons from the MPFC.

## Motor cortex layer V pyramidal neuron morphology changes early in the disease process

### LVPNs in the SOD1 motor cortex exhibit decreased dendritic arbor length from P28-P35 compared to WT controls

**Fig. 2:**
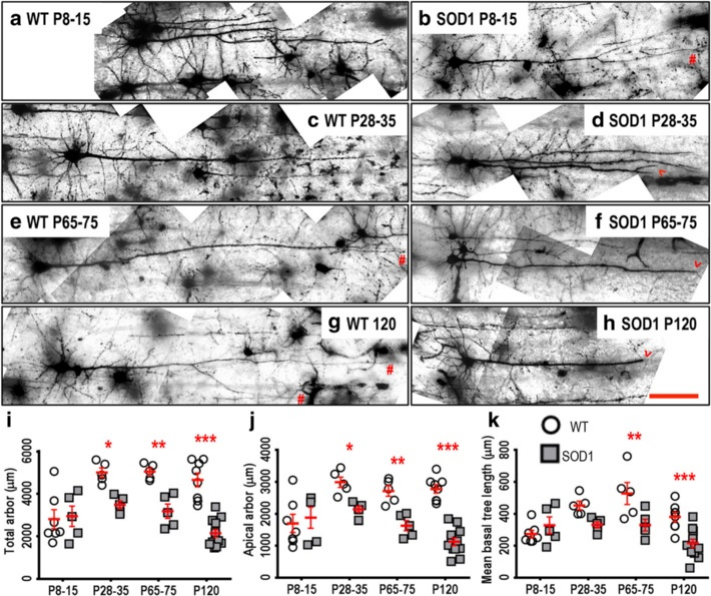
Decreased dendritic arbors of SOD1 LVPNs within the motor cortex commenced from P28-35, compared to WT controls. Images show mosaics of motor cortex LVPNs from P8-15, P28-35, P65-75 and P120 WT (**a**, **c**, **e**, **g**) and SOD1 (**b**, **d**, **f**, **h**) mice. The red hashes indicate regularly tapering apical dendrites, while the arrowheads indicate pathologic abrupt endings in SOD1 apical dendrites. **i** is a scatterplot quantifying significantly decreased total dendritic arbor length (μm) of SOD1 LVPNs (*grey squared*) compared to WT controls (*white circles*) at P28-35, P65-75 and P120. **j** is a scatterplot quantifying significantly decreased apical arbor length (μm) of SOD1 LVPNs (*grey squared*) compared to WT controls (*white circles*) at P28-35, P65-75 and P120. **k** is a scatterplot quantifying significantly decreased mean basal tree length (μm) of SOD1 LVPNs (*grey squared*) compared to WT controls (*white circles*) at P65-75 and P120. All data are mean ± SEM, with two-way ANOVAs followed by Bonferroni post-tests, *P < 0.05, **P < 0.01 and ***P < 0.001; *n* = 7, 5, 5 and 8 for WT P8-15, P28-35, P65-75 and P120 respectively; *n* = 6, 5, 5 and 12 for SOD1 P8-15, P28-35, P65-75 and P120 respectively. Scale bar: 100 μm

Motor cortex LVPN soma volume did not differ at all ages studied (Table 2). By contrast, motor cortex LVPN dendritic arbors were progressively reduced in SOD1 mice, compared to WT controls, from P28-35 onwards. While there was no difference in the total dendritic arbor length of motor cortex LVPNs in SOD1 mice versus WT controls at P8-15, there was a 30 % reduction at P28-35 (*P* = 0.016), a 37 % reduction at P65-75 (*P* = 0.0021) and a 54 % reduction at P120 (*P* = 0.0021, Table 2, Fig. 2g).

**Table 2:** Morphometric dendritic and dendritic spine parameters of LVPNs within the motor cortex. All data presented as mean ± SEM

Region	P8-15 (*n*) Neonatal	P28-35 (*n*) Pre-symptomatic	P65-75 (*n*) Onset	P120 (*n*) Mid-disease
Soma volume (μm^3^)	WT: 1374.4 ± 210.3 (*7*)	WT: 1672.7 ± 68.7 (*5*)	WT: 1709.9 ± 114.5 (*5*)	WT: 1492.5 ± 178.0 (*8*)
	SOD1: 1372.8 ± 252.9 (*5*)	SOD1: 2221.1 ± 333.7 (*5*)	SOD1: 2242.7 ± 215.8 (*5*)	SOD1: 955.3 ± 115.6 (*12*)
	Not significant	Not significant	Not significant	Not significant
Total arbor length (μm)	WT: 2808 ± 442 (*7*)	WT: 5001 ± 220 (*5*)	WT: 5046 ± 162 (*5*)	WT: 1487 ± 324 (*8*)
	SOD1: 2943 ± 474 (*5*)	SOD1: 3485 ± 179 (*5*)	SOD1: 3181 ± 336 (*5*)	SOD1: 1136 ± 181 (*12*)
	Adj. *P* > 0.99	Adj. *P* = 0.02*	Adj. *P* = 0.002**	Adj. *P* < 0.0001****
Apical arbor length (μm)	WT: 1701 ± 282 (*7*)	WT: 2991 ± 163 (*5*)	WT: 2715 ± 166 (*5*)	WT: 2781 ± 133 (*8*)
	SOD1: 1880 ± 327 (*5*)	SOD1: 2142 ± 97 (*5*)	SOD1: 1631 ± 157 (*5*)	SOD1: 1132 ± 119 (*12*)
	Adj. *P* > 0.99	Adj. *P* = 0.03*	Adj. *P* = 0.003**	Adj. *P* < 0.0001****
Basal arbor length (μm)	WT: 1113 ± 174 (*7*)	WT: 2010 ± 128 (*5*)	WT: 2131 ± 246 (*5*)	WT: 1872 ± 216 (*8*)
	SOD1: 1003 ± 163 (*5*)	SOD1: 1341 ± 103 (*5*)	SOD1: 1550 ± 189 (*5*)	SOD1: 946 ± 102 (*12*)
	Adj. *P* > 0.99	Adj. *P* = 0.07	Adj. *P* = 0.16	Adj. *P* = 0.0001***
Mean basal tree length (μm)	WT: 275 ± 22 (*7*)	WT: 452 ± 27 (*5*)	WT: 527 ± 69 (*5*)	WT: 380 ± 29 (*8*)
	SOD1: 328 ± 163 (*5*)	SOD1: 332 ± 19 (*5*)	SOD1: 330 ± 36 (*5*)	SOD1: 217 ± 29 (*12*)
	Adj. *P* > 0.99	Adj. *P* = 0.15	Adj. *P* = 0.004**	Adj. *P* = 0.0008***
Apical reach (μm)	WT: 584 ± 37 (*7*)	WT: 1059 ± 84 (*5*)	WT: 921 ± 47 (*5*)	WT: 884 ± 54 (*8*)
	SOD1: 656 ± 51 (*5*)	SOD1: 863 ± 51 (*5*)	SOD1: 580 ± 98 (*5*)	SOD1: 465 ± 32 (*12*)
	Adj. *P* > 0.99	Adj. *P* = 0.12	Adj. *P* = 0.0012**	Adj. *P* < 0.0001****
Basal reach (μm)	WT: 192 ± 19 (*7*)	WT: 321 ± 20 (*5*)	WT: 290 ± 22 (*5*)	WT: 282 ± 28 (*8*)
	SOD1: 202 ± 29 (*5*)	SOD1: 257 ± 13 (*5*)	SOD1: 254 ± 20 (*5*)	SOD1: 187 ± 28 (*12*)
	Adj. *P* > 0.99	Adj. *P* = 0.69	Adj. *P* > 0.99	Adj. *P* < 0.01*
Apical ramifications	WT: 9.7 ± 1.1 (*7*)	WT: 10.6 ± 1.1 (*5*)	WT: 11.5 ± 1.7 (*5*)	WT: 12.7 ± 1 (*8*)
	SOD1: 10.7 ± 1.6 (*5*)	SOD1: 10.6 ± 1 (*5*)	SOD1: 8.0 ± 0.5 (*5*)	SOD1: 7.5 ± 0.9 (*12*)
	Adj. *P* > 0.99	Adj. *P* > 0.99	Adj. *P* = 0.25	Adj. *P* = 0.0005***
Basal ramifications	WT: 3.8 ± 0.3 (*7*)	WT: 4.2 ± 0.2 (*5*)	WT: 4.6 ± 0.3 (*5*)	WT: 4.4 ± 0.2 (*8*)
	SOD1: 4.4 ± 0.4 (*5*)	SOD1: 3.4 ± 0.2 (*5*)	SOD1: 3.9 ± 0.2 (*5*)	SOD1: 3.4 ± 0.2 (*12*)
	Adj. *P* = 0.64	Adj. *P* = 0.31	Adj. *P* = 0.33	Adj. *P* = 0.008**
Apical spine density per 100 μm	WT: 27.7 ± 2.7 (*7*)	WT: 31.7 ± 3.1 (*5*)	WT: 33.4 ± 3 (*5*)	WT: 23.7 ± 1 (*8*)
	SOD1: 23.8 ± 4.4 (*5*)	SOD1: 17.2 ± 1.3 (*5*)	SOD1: 18.4 ± 0.8 (*5*)	SOD1: 16.4 ± 1.9 (*12*)
	Adj. *P* > 0.99	Adj. *P* = 0.002**	Adj. *P* = 0.002**	Adj. *P* = 0.049*
Basal spine density per 100 μm	WT: 21.2 ± 1.7 (*7*)	WT: 27.2 ± 3 (*5*)	WT: 29.9 ± 2.9 (*5*)	WT: 19.5 ± 0.8 (*8*)
	SOD1: 13.7 ± 5.7 (*5*)	SOD1: 13.8 ± 1.2 (*5*)	SOD1: 15.9 ± 2 (*5*)	SOD1: 13.7 ± 0.8 (*12*)
	Adj. *P* = 0.10	Adj. *P* = 0.002**	Adj. *P* = 0.001**	Adj. *P* = 0.11

All analyses were unpaired two-way ANOVAs for age and genotype of LVPNs from the motor cortex comparing soma volume (Age: *P* = 0.0002***; Genotype: *P* = 0.3378), total dendritic arbor length (Age: *P* = 0.0004***; Genotype: *P* < 0.0001****), apical dendritic arbor length (Age: *P* = 0.0029**; Genotype: *P* < 0.0001****), basal dendritic arbor length (Age: *P* = 0.0002***; Genotype: *P* < 0.0001****), mean basal dendritic tree length (Age: *P* = 0.0009***; Genotype: *P* = 0.0001***), apical dendritic reach (Age: *P* < 0.0001****; Genotype: *P* < 0.0001****), basal dendritic reach (Age: *P* = 0.0142*; Genotype: *P* = 0.0251*), apical branch ramifications (Age: *P* = 0.9265; Genotype: *P* = 0.0270*), basal branch ramifications (Age: *P* = 0.3522; Genotype: *P* = 0.0153*), apical dendritic spine density per 100 μm (Age: *P* = 0.0350*; Genotype: *P* < 0.0001****) and basal dendritic spine density per 100 μm (Age: *P* = 0.0287*; Genotype: *P* < 0.0001****). Bonferroni post-tests were used to compare the effect of genotype at each age group, with the adjusted *P*-value (adj. *P*) reported in the table for parameters that had significant genotype effects. Each animal used (*n*) contains mean values from a minimum of 3 individual neurons, specifically P8-15 (WT: 21 neurons; SOD1: 15 neurons), P28-35 (WT: 22 neurons; SOD1: 21 neurons), P65-75 (WT: 16 neurons; SOD1: 16 neurons) and P120 (WT: 29 neurons; SOD1: 38 neurons)

There was a similar progressive reduction in the apical arbor length of motor cortex LVPNs of SOD1 mice, compared to WT controls, from P28-35 onwards. While apical arbor length was not altered at P8-15, there was a 28 % reduction at P28-35 (*P* = 0.03), a 40 % reduction at P65-75 (*P* = 0.0034) and a 59 % reduction at P120 (*P* < 0.0001, Table 2, Fig. 2h). The apical reach of motor cortex LVPNs in SOD1 and WT control mice was similar at P8-15 or P28-35, but then decreased by 37 % at P65-75 (*P* = 0.0012) and by 47 % at P120 in SOD1 mice, compared to WT controls (*P* < 0.0001, Table 2). The apical ramifications of motor cortex LVPNs in SOD1 mice were also unchanged at P8-15, P28-35, or P65-75, but there was a 40 % reduction at P120 in SOD1 mice, compared to WT controls (*P* = 0.0012**, Table 2).

By contrast, the basal dendritic arbor did not change until later in the disease process. The basal arbor length of motor cortex LVPNs of SOD1 mice was unchanged at P8-15, P28-35 or P65-75 (Table 2), but by P120, there was a 47 % reduction in motor cortex LVPN basal arbor length in of SOD1 mice, compared to WT controls (*P* = 0.0001, Table 2). Similarly, the mean basal tree length of motor cortex LVPNs in SOD1 mice was not different at P8-15 or P28-35, but a 37 % reduction was seen at P65-75 (*P* = 0.004) and a 35 % reduction was present at P120 in SOD1 mice, compared to WT controls (*P* = 0.0008, Table 2, Fig. 2i). There was no difference in the basal reach of motor cortex LVPNs in SOD1 mice at P8-15, P28-35 or P65-75 (Table 2), until P120, when there was a 34 % reduction in SOD1 mice, compared to WT controls (*P* < 0.0001, Table 2). The basal ramifications of motor cortex LVPNs in SOD1 mice were also unchanged at P8-15, P28-35 or P65-75 (Table 2), but at P120, there was a 23 % reduction in SOD1 mice, compared to WT controls (*P* = 0.0012, Table 2).

Taken together, these data suggest that motor cortex LVPN dendritic regression is an early pathogenic occurrence in SOD1 mice, with apical arbor length shortening from P28-35 onwards, some 4–6 weeks before symptom onset and upper MN loss in this strain [29, 35, 45]. Regression of basal dendrites occurs later, with shortening of basal tree length beginning at P65-75, and decreased total basal arbor length by P120. By mid-disease (P120), neuronal complexity (ramifications) of apical and basal dendrites is reduced, compared to WT controls. These changes in the dendritic arbors of LVPNs from the motor cortex of SOD1 mice are likely to significantly alter the passive or active dendritic integration of synaptic inputs to these neurons. As the dendritic spine is the physical postsynaptic substrate for neuronal synaptic inputs in pyramidal neurons [59] and a prime site of excitotoxic neuronal degeneration in many neuropathologies, including ALS [30, 39, 43, 44, 57, 65], we next quantified the dendritic spine density of LVPNs in the motor cortex.

### Motor cortex LVPNs in SOD1 mice show decreased apical and basal dendritic spine density from P28-P35 onwards

**Fig. 3:**
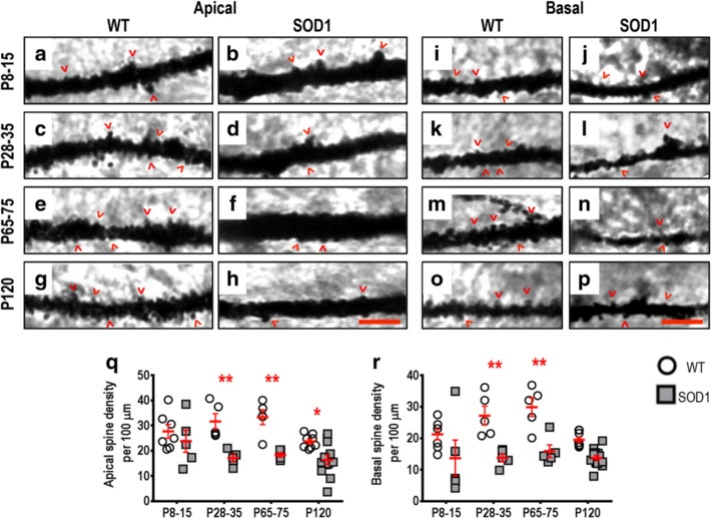
Decreased apical and basal dendritic spine density of SOD1 LVPNs within the motor cortex commenced from P28-35, compared to WT controls. High-magnification images of apical dendrites from P8-15, P28-35, P65-75 and P120 WT (**a**, **c**, **e**, **g**) and SOD1 (**b**, **d**, **f**, **h**) motor cortex are shown. High-magnification images of basal dendrites from P8-15, P28-35, P65-75 and P120 WT (**i**, **k**, **m**, **o**) and SOD1 (**j**, **l**, **n**, **p**) motor cortex are also shown. Examples of apical and basal dendritic spines are identified with red arrowheads. **q** is a scatterplot quantifying significantly decreased apical dendritic spine density per 100 μm of SOD1 LVPNs (*grey squared*) compared to WT controls (*white circles*) at P28-35, P65-75 and P120. **r** is a scatterplot quantifying significantly decreased basal dendritic spine density per 100 μm of SOD1 LVPNs (*grey squared*) compared to WT controls (*white circles*) at P28-35 and P65-75. All data are mean ± SEM, with two-way ANOVAs followed by Bonferroni post-tests, *P < 0.05 and **P < 0.01; *n* = 7, 5, 5 and 8 for WT P8-15, P28-35, P65-75 and P120 respectively; *n* = 6, 5, 5 and 12 for SOD1 P8-15, P28-35, P65-75 and P120 respectively. Scale bar: **h** and **p**, 3 μm

The dendritic arbors of SOD1 LVPNs within the motor cortex had significantly lower apical (Fig. 3a–h) and basal (Fig. 3i–p) spine densities, compared to WT controls. There were no differences in either apical or basal dendritic spine density of motor cortex LVPNs in SOD1 mice at P8-15 (Table 2). At P28-35, apical spine density was reduced by 46 % (*P* = 0.0021, Table 2, Fig. 3q) and basal spine density was similarly decreased by 40 % (*P* = 0.0017, Table 2, Fig. 3r). This reduction persisted at P65-75, where there was a 45 % reduction in the apical dendritic spine density (*P* = 0.0015, Table 2, Fig. 3q) and a 47 % reduction in the basal dendritic spine density (*P* = 0.0010, Table 2, Fig. 3r). By P120, apical spine density was still reduced by 31 % (*P* = 0.0429*, Table 2, Fig. 3q), but basal spine density was not different to WT control levels (Table 2, Fig. 3r).

Taken together, these results provide strong morphological evidence for a profound alteration in the synaptic inputs to both apical and basal dendrites of LVPNs within the motor cortex from pre-symptomatic ages (P28-35).

The concurrent loss of upper MNs (including LVPNs of the motor cortex) and lower MNs is pathognomonic for ALS, but whether other motor cortex pyramidal neurons are also affected is relatively unknown. We thus investigated whether the structural changes observed in LVPNs were recapitulated in layer II/III pyramidal neurons in the motor cortex.

### Motor cortex LII-IIIPNs in SOD1 mice also show early decreases in apical spine density and late decreases in apical dendritic arbor length

**Fig. 4:**
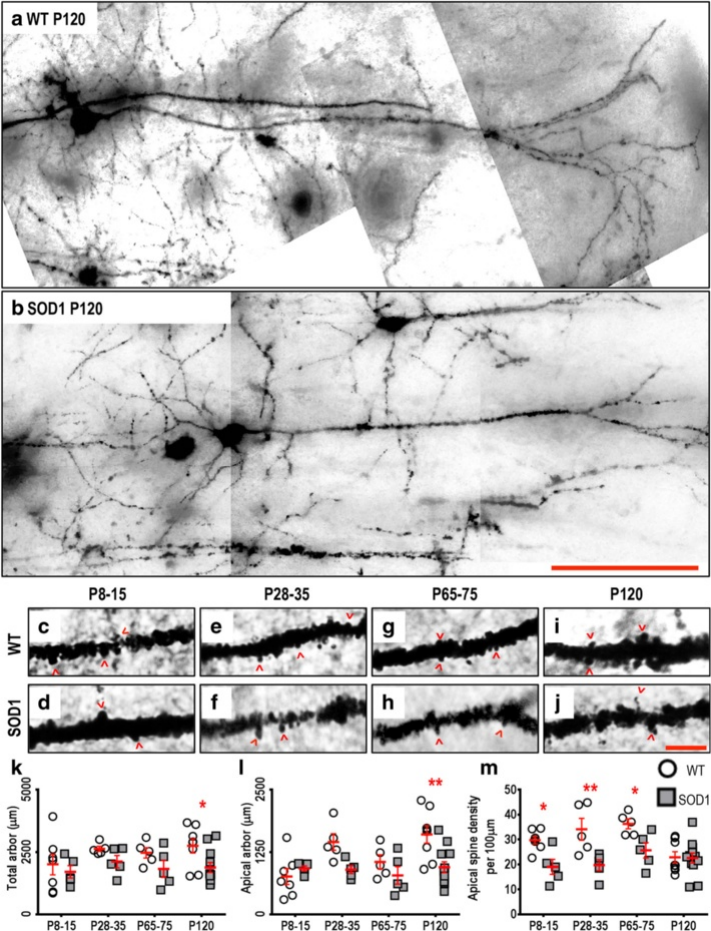
Decreased dendritic arbors of SOD1 LII-IIIPNs within the motor cortex at P120 and decreased apical spine density commenced from P8-15, compared to WT controls. Images show mosaics of motor cortex LII-IIIPNs from P120 WT (**a**) and SOD1 (**b**) mice. High-magnification images of apical dendrites from P8-15, P28-35, P65-75 and P120 WT (**c**, **e**, **g**, **i**) and SOD1 (**d**, **f**, **h**, **j**) motor cortex, with examples of dendritic spines indicated with open arrowheads, are also shown. **k** is a scatterplot quantifying significantly decreased total dendritic arbor length (μm) of SOD1 LII-IIIPNs (*grey squared*) compared to WT controls (*white circles*) at P120. **l** is a scatterplot quantifying significantly decreased apical arbor length (μm) of SOD1 LII-IIIPNs (*grey squared*) compared to WT controls (*white circles*) at P120. **m** is a scatterplot quantifying significantly decreased apical dendritic spine density per 100 μm of SOD1 LII-IIIPNs (*grey squared*) compared to WT controls (*white circles*) at P8-15, P28-35 and P65-75. All data are mean ± SEM, with two-way ANOVAs followed by Bonferroni post-tests, *P < 0.05, **P < 0.01 and ***P < 0.001; *n* = 7, 5, 5 and 8 for WT P8-15, P28-35, P65-75 and P120 respectively; *n* = 6, 5, 5 and 12 for SOD1 P8-15, P28-35, P65-75 and P120 respectively. Scale bar: **b**, 100 μm; **j**, 5 μm

As for LVPNs, the soma volumes of motor cortex LII-IIIPNs in SOD1 mice were unchanged at all ages studied (Table 3). In addition, there were no differences in the total arbor length or apical arbor length of motor cortex LII-IIIPNs in SOD1 mice, compared to WT controls, at P8-15, P28-35 or P65-75 (Table 3, Fig. 4k, 4l, 4m). At P120, the total dendritic arbor length was decreased by 31 % (*P* = 0.016, Table 3, Fig. 4k) and the apical arbor length was reduced by 41 % in motor cortex LII-IIIPNs in SOD1 mice (*P* = 0.0014, Table 3, Fig. 4l). No other measures of dendritic structure of motor cortex LII-IIIPNs (basal arbor length, mean basal tree length, apical reach, basal reach, apical ramifications or basal ramifications) were significantly altered in SOD1 mice at any age studied (Table 3). These data show that, in contrast to the early dendritic regression seen in motor cortex LVPNs, LII-IIIPN dendritic shortening occurs much later in SOD1 mice, at ages when upper MN loss has already occurred and motor control is obviously disrupted. In addition, dendritic shortening was confined to the apical dendrites, as basal dendritic trees of LII-IIIPNs did not show any significant differences at any age.

**Table 3:** Morphometric dendritic and dendritic spine parameters of LII-IIIPNs within the motor cortex. All data presented as mean ± SEM

Region	P8-15 (*n*) Neonatal	P28-35 (*n*) Pre-symptomatic	P65-75 (*n*) Onset	P120 (*n*) Mid-disease
Soma volume (μm^3^)	WT: 587.5 ± 98.1 (*7*)	WT: 1338.5 ± 119.3 (*5*)	WT: 1297.5 ± 194.5 (*5*)	WT: 998.3 ± 112.4 (*8*)
	SOD1: 997.0 ± 266.0 (*5*)	SOD1: 1063.1 ± 271.3 (*5*)	SOD1: 1218.4 ± 356.2 (*5*)	SOD1: 701.9 ± 101.4 (*12*)
	Not significant	Not significant	Not significant	Not significant
Total arbor length (μm)	WT: 2005 ± 414 (*7*)	WT: 2626 ± 101 (*5*)	WT: 2468 ± 208 (*5*)	WT: 2747 ± 288 (*8*)
	SOD1: 1709 ± 244 (*5*)	SOD1: 2116 ± 241 (*5*)	SOD1: 1831 ± 338 (*5*)	SOD1: 1883 ± 196 (*12*)
	Adj. *P* > 0.99	Adj. *P* > 0.99	Adj. *P* = 0.67	Adj. *P* < 0.047*
Apical arbor length (μm)	WT: 762 ± 159 (*7*)	WT: 1452 ± 165 (*5*)	WT: 1052 ± 141 (*5*)	WT: 1602 ± 176 (*8*)
	SOD1: 922 ± 47 (*5*)	SOD1: 899 ± 77 (*5*)	SOD1: 785 ± 176 (*5*)	SOD1: 947 ± 107 (*12*)
	Adj. *P* > 0.99	Adj. *P* = 0.09	Adj. *P* > 0.99	Adj. *P* < 0.001**
Basal arbor length (μm)	WT: 1242 ± 268 (*7*)	WT: 1174 ± 167 (*5*)	WT: 1415 ± 77 (*5*)	WT: 1094 ± 174 (*8*)
	SOD1: 787 ± 219 (*5*)	SOD1: 1208 ± 202 (*5*)	SOD1: 1046 ± 164 (*5*)	SOD1: 963 ± 104 (*12*)
	Not significant	Not significant	Not significant	Not significant
Mean basal tree length (μm)	WT: 300 ± 57 (*7*)	WT: 290 ± 43 (*5*)	WT: 314 ± 24 (*5*)	WT: 320 ± 47 (*8*)
	SOD1: 225 ± 58 (*5*)	SOD1: 317 ± 44 (*5*)	SOD1: 283 ± 27 (*5*)	SOD1: 257 ± 27 (*12*)
	Not significant	Not significant	Not significant	Not significant
Apical reach (μm)	WT: 303 ± 28 (*7*)	WT: 599 ± 48 (*5*)	WT: 482 ± 65 (*5*)	WT: 450 ± 48 (*8*)
	SOD1: 424 ± 74 (*5*)	SOD1: 443 ± 29 (*5*)	SOD1: 398 ± 64 (*5*)	SOD1: 321 ± 37 (*12*)
	Not significant	Not significant	Not significant	Not significant
Basal reach (μm)	WT: 199 ± 22 (*7*)	WT: 209 ± 21 (*5*)	WT: 249 ± 18 (*5*)	WT: 205 ± 18 (*8*)
	SOD1: 178 ± 34 (*5*)	SOD1: 229 ± 29 (*5*)	SOD1: 193 ± 26 (*5*)	SOD1: 188 ± 26 (*12*)
	Not significant	Not significant	Not significant	Not significant
Apical ramifications	WT: 5.2 ± 0.7 (*7*)	WT: 5.7 ± 0.6 (*5*)	WT: 5.9 ± 0.3 (*5*)	WT: 7.9 ± 0.7 (*8*)
	SOD1: 8.5 ± 0.8 (*5*)	SOD1: 5.3 ± 0.3 (*5*)	SOD1: 5.7 ± 1 (*5*)	SOD1: 6.3 ± 0.4 (*12*)
	Not significant	Not significant	Not significant	Not significant
Basal ramifications	WT: 3.9 ± 0.5 (*7*)	WT: 3.7 ± 0.2 (*5*)	WT: 3.9 ± 0.2 (*5*)	WT: 4.3 ± 0.3 (*8*)
	SOD1: 3.5 ± 0.3 (*5*)	SOD1: 4.8 ± 0.4 (*5*)	SOD1: 3.4 ± 0.3 (*5*)	SOD1: 3.9 ± 0.2 (*12*)
	Not significant	Not significant	Not significant	Not significant
Apical spine density per 100 μm	WT: 29.8 ± 1.6 (*7*)	WT: 34.2 ± 4.4 (*5*)	WT: 36.3 ± 2 (*5*)	WT: 22.9 ± 2.3 (*8*)
	SOD1: 19.8 ± 3 (*5*)	SOD1: 19.7 ± 2.2 (*5*)	SOD1: 25.7 ± 3.1 (*5*)	SOD1: 22.6 ± 2 (*12*)
	Adj. *P* = 0.0277*	Adj. *P* = 0.0039**	Adj. *P* = 0.0494*	Adj. *P* > 0.9999
Basal spine density per 100 μm	WT: 17.4 ± 1.8 (*7*)	WT: 26.7 ± 3.2 (*5*)	WT: 27.5 ± 3.5 (*5*)	WT: 17.2 ± 2.5 (*8*)
	SOD1: 20.9 ± 4.3 (*5*)	SOD1: 16.5 ± 2.1 (*5*)	SOD1: 19.6 ± 1.7 (*5*)	SOD1: 18.3 ± 1.9 (*12*)
	Not significant	Not significant	Not significant	Not significant

All analyses were unpaired two-way ANOVAs for age and genotype of LII-IIIPNs from the motor cortex comparing soma volume (Age: *P* = 0.0272*; Genotype: *P* = 0.6421), total dendritic arbor length (Age: *P* = 0.3006; Genotype: *P* = 0.0082**), apical dendritic arbor length (Age: *P* = 0.0185*; Genotype: *P* = 0.0038**), basal dendritic arbor length (Age: *P* = 0.5556; Genotype: *P* = 0.0889), mean basal dendritic tree length (Age: *P* = 0.8449; Genotype: *P* = 0.2142), apical dendritic reach (Age: *P* = 0.0218*; Genotype: *P* = 0.0963), basal dendritic reach (Age: *P* = 0.5799; Genotype: *P* = 0.3441), apical branch ramifications (Age: *P* = 0.0311*; Genotype: *P* = 0.1325), basal branch ramifications (Age: *P* = 0.2204; Genotype: *P* = 0.8882), apical dendritic spine density per 100 μm (Age: *P* = 0.0155*; Genotype: *P* < 0.0001****) and basal dendritic spine density per 100 μm (Age: *P* = 0.1380; Genotype: *P* = 0.0877). Bonferroni post-tests were used to compare the effect of genotype at each age group, with the adjusted *P*-value (adj. *P*) reported in the table for parameters that had significant genotype effects. Each animal used (*n*) contains mean values from a minimum of 2 individual neurons, specifically P8-15 (WT: 20 neurons; SOD1: 10 neurons), P28-35 (WT: 11 neurons; SOD1: 11 neurons), P65-75 (WT: 11 neurons; SOD1: 13 neurons) and P120 (WT: 21 neurons; SOD1: 25 neurons)

We also quantified the dendritic spine density in apical and basal arbors of motor cortex LII-IIIPNs. Intriguingly, apical dendritic spine densities were significantly lower in motor cortex LII-IIIPNs for all but the oldest age group (Fig. 4c–j). Apical spine density was reduced by 36 % (*P* = 0.0039, Table 3, Fig. 4m), 42 % (*P* = 0.0039, Table 3, Fig. 4m) and 29 % (*P* = 0.049, Table 3, Fig. 4m) at P8-15, P28-35 and P65-75 ages, respectively, but at P120, WT apical spine density declined to SOD1 levels (Table 3). By contrast, basal dendrite spine density did not significantly differ between SOD1 and WT motor cortex LII-IIIPNs. These unexpected and novel results provide evidence for an early and significant alteration in the synaptic inputs to apical dendrites of LII-IIIPNs within the SOD1 motor cortex. Taken together with our observation of a later decrease in apical and total dendritic arbor lengths in LII-IIIPNs in the SOD1 motor cortex, these data suggest that early neuronal pathology may occur in cortical populations other than motor cortex LVPNs. Although ALS patients with SOD1 mutation do not typically show prominent cognitive deficits, cognitive deficits are a prominent feature in some ALS patients [18, 37, 38, 47], and pyramidal neurons in the MPFC, a key cortical area for cognitive function, are altered in SOD1 mutant mice at P60 [57]. We therefore next investigated whether pyramidal neurons of the MPFC showed early changes in dendritic structures and spine density.

### MPFC pyramidal neurons within the exhibit altered basal dendritic arbor lengths from P8-P15 and decreased spine density from P28-35

**Fig. 5:**
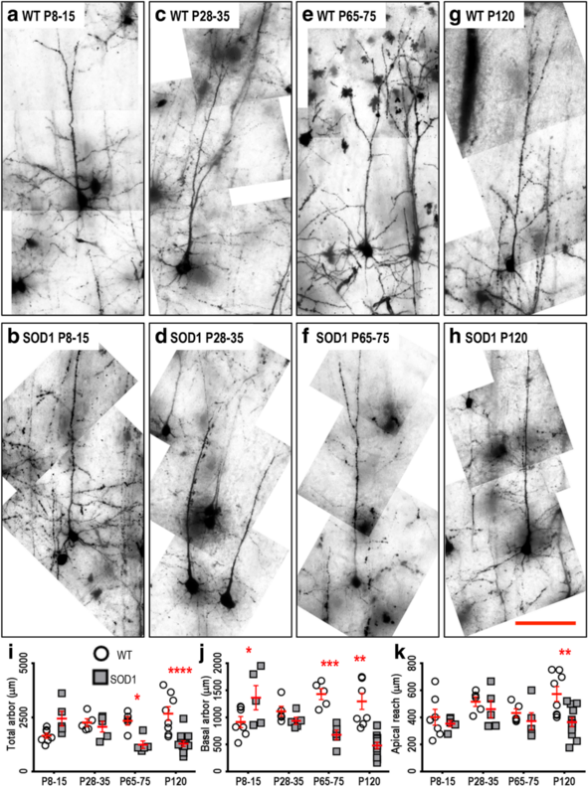
Altered dendritic arbors of SOD1 pyramidal neurons within the MPFC commenced from P8-15, compared to WT controls. Images show mosaics of MPFC pyramidal neurons from P8-15, P28-35, P65-75 and P120 WT (**a**, **c**, **e**, **g**) and SOD1 (**b**, **d**, **f**, **h**) mice. **i** is a scatterplot quantifying significantly decreased total dendritic arbor length (μm) of SOD1 pyramidal neurons (*grey squared*) compared to WT controls (*white circles*) at P65-75 and P120. **j** is a scatterplot quantifying significantly increased basal arbor length (μm) of SOD1 pyramidal neurons (grey squared) compared to WT controls (*white circles*) at P8-16 and a significant decrease compared to WT at P65-75 and P120. **k** is a scatterplot quantifying significantly decreased apical dendritic reach (μm) of SOD1 pyramidal neurons (*grey squared*) compared to WT controls (*white circles*) at P120. All data are mean ± SEM, with two-way ANOVAs followed by Bonferroni post-tests, *P < 0.05, **P < 0.01, ***P < 0.001 and ****P < 0.0001; *n* = 7, 5, 5 and 8 for WT P8-15, P28-35, P65-75 and P120 respectively; *n* = 6, 5, 5 and 12 for SOD1 P8-15, P28-35, P65-75 and P120 respectively. Scale bar: 50 μm

As for motor cortex pyramidal neurons, the soma volume of MPFC pyramidal neurons in SOD1 mice did not change, compared to WT controls (Table 4). By contrast, the dendritic arbors of MPFC pyramidal neurons in SOD1mice were significantly altered from P8-15 onwards, with the earliest changes appearing in basal dendrites (Fig. 5). The total dendritic arbor length of MPFC pyramidal neurons was reduced by 47 % at P65-75 (*P* = 0.0142, Table 4, Fig. 5i) and reduced by 52 % at P120 (*P* < 0.0001, Table 4, Fig. 5i). Although the apical arbor length of MPFC pyramidal neurons did not change at any age studied (Table 4), there was a 36 % reduction in apical reach at P120 (*P* = 0.0022, Table 4, Fig. 5k), consistent with MPFC cortical thinning at the same age. Initially, both total basal arbor length and basal tree length of MPFC pyramidal neurons in SOD1 mice were increased at P8-15, by 49 % for arbor length (*P* = 0.048, Table 4, Fig. 5j) and by 82 % for tree length (*P* = 0.024, Table 4, Fig. 5j). The increase in both of these basal dendrite parameters subsequently regressed; there was no difference between SOD1 and WT control mice at P28-35 (Table 4, Fig. 5j), followed by a 53 % reduction in basal arbor length (*P* = 0.0008, Table 4, Fig. 5j) and 45 % reduction in basal tree length (*P* = 0.023, Table 4) at P65-75 and then a further 63 % reduction in basal arbor length (*P* < 0.0001, Table 4, Fig. 5j) and a 65 % reduction in basal tree length (adj. *P* < 0.0001****, Table 4) at P120. There were no differences between genotypes in the basal reach, apical ramification or basal ramification of MPFC pyramidal neurons at any age studied (Table 4).

**Table 4:** Morphometric dendritic and dendritic spine parameters of pyramidal neurons within the MPFC. All data presented as mean ± SEM

Region	P8-15 (*n*) Neonatal	P28-35 (*n*) Pre-symptomatic	P65-75 (*n*) Onset	P120 (*n*) Mid-disease
Soma volume (μm^3^)	WT: 951.8 ± 263.4 (*7*)	WT: 1337.9 ± 150.1 (*5*)	WT: 1215.4 ± 205.2 (*5*)	WT: 860.1 ± 112.3 (*8*)
	SOD1: 1337.1 ± 216.9 (*5*)	SOD1: 1236.3 ± 267.4 (*5*)	SOD1: 1203.6 ± 266.1 (*5*)	SOD1: 773.5 ± 132.6 (*12*)
	Not significant	Not significant	Not significant	Not significant
Total arbor length (μm)	WT: 1658 ± 119 (*7*)	WT: 2263 ± 175 (*5*)	WT: 2343 ± 190 (*5*)	WT: 2675 ± 321 (*8*)
	SOD1: 2446 ± 336 (*5*)	SOD1: 2074 ± 238 (*5*)	SOD1: 1243 ± 170 (*5*)	SOD1: 1294 ± 133 (*12*)
	Adj. *P* = 0.09	Adj. *P* > 0.99	Adj. *P* = 0.01*	Adj. *P* < 0.0001****
Apical arbor length (μm)	WT: 783 ± 145 (*7*)	WT: 1152 ± 84 (*5*)	WT: 912 ± 125 (*5*)	WT: 1378 ± 191 (*8*)
	SOD1: 1081 ± 204 (*5*)	SOD1: 1137 ± 221 (*5*)	SOD1: 564 ± 119 (*5*)	SOD1: 810 ± 197 (*12*)
	Not significant	Not significant	Not significant	Not significant
Basal arbor length (μm)	WT: 915 ± 100 (*7*)	WT: 1111 ± 175 (*5*)	WT: 1430 ± 102 (*5*)	WT: 1296 ± 149 (*8*)
	SOD1: 1365 ± 225 (*5*)	SOD1: 937 ± 238 (*5*)	SOD1: 879 ± 88 (*5*)	SOD1: 484 ± 60 (*12*)
	Adj. *P* = 0.048*	Adj. *P* > 0.99	Adj. *P* = 0.0008***	Adj. *P* < 0.0001****
Mean basal tree length (μm)	WT: 233 ± 24 (*7*)	WT: 305 ± 9 (*5*)	WT: 350 ± 55 (*5*)	WT: 381 ± 32 (*8*)
	SOD1: 424 ± 93 (*5*)	SOD1: 273 ± 27 (*5*)	SOD1: 190 ± 17 (*5*)	SOD1: 133 ± 15 (*12*)
	Adj. *P* = 0.004**	Adj. *P* > 0.99	Adj. *P* = 0.03*	Adj. *P* < 0.0001****
Apical reach (μm)	WT: 402 ± 58 (*7*)	WT: 517 ± 35 (*5*)	WT: 432 ± 32 (*5*)	WT: 573 ± 55 (*8*)
	SOD1: 355 ± 21 (*5*)	SOD1: 460 ± 63 (*5*)	SOD1: 372 ± 61 (*5*)	SOD1: 365 ± 32 (*12*)
	Adj. *P* > 0.99	Adj. *P* > 0.99	Adj. *P* > 0.99	Adj. *P* < 0.002**
Basal reach (μm)	WT: 207 ± 28 (*7*)	WT: 226 ± 25 (*5*)	WT: 248 ± 15 (*5*)	WT: 216 ± 14 (*8*)
	SOD1: 302 ± 56 (*5*)	SOD1: 192 ± 7 (*5*)	SOD1: 148 ± 20 (*5*)	SOD1: 135 ± 23 (*12*)
	Not significant	Not significant	Not significant	Not significant
Apical ramifications	WT: 5.3 ± 0.8 (*7*)	WT: 5.9 ± 0.6 (*5*)	WT: 5.8 ± 0.4 (*5*)	WT: 6.9 ± 0.8 (*8*)
	SOD1: 7.5 ± 0.6 (*5*)	SOD1: 6.6 ± 0.9 (*5*)	SOD1: 5.5 ± 1.5 (*5*)	SOD1: 5.7 ± 0.8 (*12*)
	Not significant	Not significant	Not significant	Not significant
Basal ramifications	WT: 3.2 ± 0.3 (*7*)	WT: 4.2 ± 0.5 (*5*)	WT: 4 ± 0.3 (*5*)	WT: 4.4 ± 0.2 (*8*)
	SOD1: 4.4 ± 0.3 (*5*)	SOD1: 4.2 ± 0.1 (*5*)	SOD1: 3.4 ± 0.4 (*5*)	SOD1: 3.1 ± 0.3 (*12*)
	Not significant	Not significant	Not significant	Not significant
Apical spine density per 100 μm	WT: 21.1 ± 2.7 (*7*)	WT: 25.3 ± 1.1 (*5*)	WT: 33.8 ± 1.6 (*5*)	WT: 27.5 ± 1.6 (*8*)
	SOD1: 21 ± 3.4 (*5*)	SOD1: 20 ± 2.8 (*5*)	SOD1: 15.4 ± 2.5 (*5*)	SOD1: 19.6 ± 2.5 (*12*)
	Adj. *P* > 0.9999	Adj. *P* = 0.8341	Adj. *P* = 0.0002***	Adj. *P* = 0.0415*
Basal spine density per 100 μm	WT: 15.9 ± 1.7 (*7*)	WT: 22.4 ± 2.2 (*5*)	WT: 26.2 ± 1.6 (*5*)	WT: 22.1 ± 1.8 (*8*)
	SOD1: 18.4 ± 1.2 (*5*)	SOD1: 12.5 ± 1.4 (*5*)	SOD1: 14.3 ± 2.5 (*5*)	SOD1: 14.2 ± 1.6 (*12*)
	Adj. *P* > 0.99	Adj. *P* = 0.01*	Adj. *P* = 0.002**	Adj. *P* = 0.004**

All analyses were unpaired two-way ANOVAs for age and genotype of pyramidal neurons from the MPFC comparing soma volume (Age: *P* = 0.0637; Genotype: *P* = 0.7515), total dendritic arbor length (Age: *P* = 0.5093; Genotype: *P* = 0.0063**), apical dendritic arbor length (Age: *P* = 0.0763; Genotype: *P* = 0.1713), basal dendritic arbor length (Age: *P* = 0.1392; Genotype: *P* = 0.0005***), mean basal dendritic tree length (Age: *P* = 0.2178; Genotype: *P* = 0.0238*), apical dendritic reach (Age: *P* = 0.1008; Genotype: *P* = 0.0122*), basal dendritic reach (Age: *P* = 0.0267*; Genotype: *P* = 0.1295), apical branch ramifications (Age: *P* = 0.8608; Genotype: *P* = 0.5692), basal branch ramifications (Age: *P* = 0.4943; Genotype: *P* = 0.4028), apical dendritic spine density per 100 μm (Age: *P* = 0.6262; Genotype: *P* = 0.0001***) and basal dendritic spine density per 100 μm (Age: *P* = 0.4772; Genotype: *P* < 0.0001****). Bonferroni post-tests were used to compare the effect of genotype at each age group, with the adjusted *P*-value (adj. *P*) reported in the table for parameters that had significant genotype effects. Each animal used (*n*) contains mean values from multiple individual neurons, specifically P8-15 (WT: 12 neurons; SOD1: 10 neurons), P28-35 (WT: 10 neurons; SOD1: 10 neurons), P65-75 (WT: 10 neurons; SOD1: 13 neurons) and P120 (WT: 16 neurons; SOD1: 20 neurons)

**Fig. 6:**
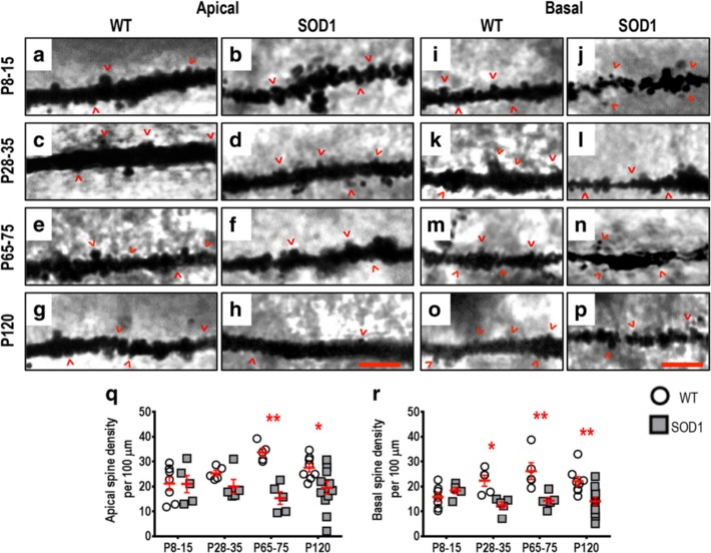
Decreased apical and basal dendritic spine density of SOD1 pyramidal neurons within the MPFC commenced from P28-35, compared to WT controls. High-magnification images of apical dendrites from P8-15, P28-35, P65-75 and P120 WT (**a**, **c**, **e**, **g**) and SOD1 (**b**, **d**, **f**, **h**) MPFC are shown. High-magnification images of basal dendrites from P8-15, P28-35, P65-75 and P120 WT (**i**, **k**, **m**, **o**) and SOD1 (**j**, **l**, **n**, **p**) motor cortex are also shown. Examples of apical and basal dendritic spines are identified with red arrowheads. **q** is a scatterplot quantifying significantly decreased apical dendritic spine density per 100 μm of SOD1 LVPNs (*grey squared*) compared to WT controls (*white circles*) at P65-75 and P120. **r** is a scatterplot quantifying significantly decreased basal dendritic spine density per 100 μm of SOD1 LVPNs (*grey squared*) compared to WT controls (*white circles*) at P28-35, P65-75 and P120. All data mean ± SEM, with two-way ANOVAs followed by Bonferroni post-tests, *P < 0.05 and **P < 0.01. *n* = 7, 5, 5 and 8 for WT P8-15, P28-35, P65-75 and P120 respectively. *n* = 6, 5, 5 and 12 for SOD1 P8-15, P28-35, P65-75 and P120 respectively. Scale bar: **h** and **p**, 5 μm

The dendritic arbors of SOD1 pyramidal neurons within the MPFC had significantly decreased apical (Fig. 6a–h) and basal (Fig. 6i–p) spine densities. The apical dendritic spine density of MPFC pyramidal neurons in SOD1 mice were unchanged at P8-15 or P28-35, but then decreased by 54 % (*P* = 0.0002) at P65-75, and 29 % (*P* = 0.042) at P120 (Table 4, Fig. 6q). Although we observed a marked increase in basal arbor length at P8-15, there was no difference in the basal dendritic spine density of MPFC pyramidal neurons in SOD1 mice at this age (Table 4, Fig. 6r). However, basal dendritic spine density was then decreased at later ages; by 44 % at P28-35 (*P* = 0.011); by 46 % at P65-75 (*P* = 0.0017); and by 36 % at P120 (*P* = 0.042, Table 4, Fig. 6r).

Taken together, these changes in MPFC pyramidal neuron dendrites and spine densities indicate that extensive and early changes occur in the MPFC of SOD1 mice, suggesting a neurobiological basis for both motor and cognitive dysfunction in ALS. In contrast to the early dendritic regression of apical dendrites seen in motor cortex, MPFC pyramidal neurons showed an initial increase in their basal dendritic arbor, followed by regression of basal dendrites, so that by P65-75, their basal arbor was decreased, consistent with a previous study [57]. As apical arbor length did not change significantly, the decrease in total arbor length apparent from P65-75 must be largely due to selective regression of basal dendrites, although apical dendrite reach did decrease by P120, contributing to MPFC cortical thinning at P120 (see results above and Table 1). The extensive nature of the changes in MPFC pyramidal neurons led us to investigate whether there were alterations in pyramidal neurons in cortical areas whose function is usually thought to be intact in ALS, such as somatosensory cortex. As motor cortex showed changes in both LVPNs and LII-IIIPNs, we next assessed whether LVPNs and LII-IIPNs of the somatosensory cortex in SOD1 mice showed changes in dendritic arbor and dendritic spine density [13, 23].

### Somatosensory cortex LVPNs in SOD1 mice show decreased apical and basal dendritic spine density from P28-P35, but no changes in their dendritic arbors

**Fig. 7:**
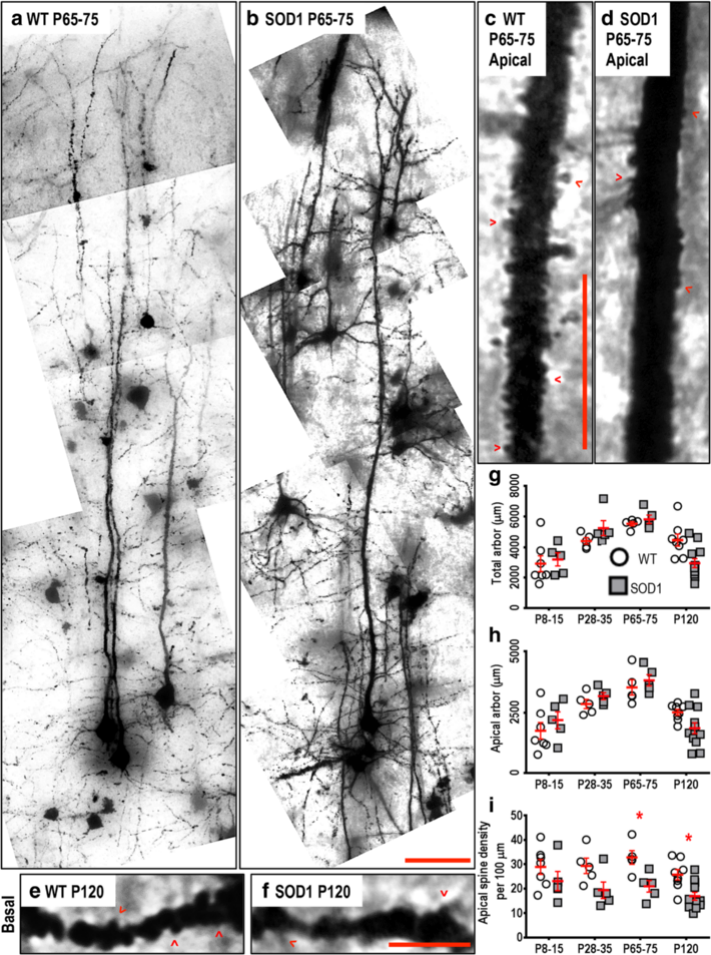
Decreased apical dendritic spine density of SOD1 LVPNs within the somatosensory cortex commencing from P65-75, compared to WT controls. Images show mosaics of somatosensory cortex LVPNs from P65-75 WT (**a**) and SOD1 (**b**) mice. High-magnification images of apical dendrites from P65-75 WT (**c**), SOD1 (**d**) and basal dendrites from P120 WT (**e**) and SOD1 (**f**) mice somatosensory cortex are also shown. Examples of apical and basal dendritic spines are identified with red arrowheads. **g** shows a scatterplot quantifying unchanged total dendritic arbor length (μm) of SOD1 LVPNs (*grey squared*) compared to WT controls (*white circles*). **h** shows a scatterplot quantifying unchanged apical arbor length (μm) of SOD1 LVPNs (*grey squared*) compared to WT controls (*white circles*). **i** shows a scatterplot quantifying significantly decreased apical dendritic spine density per 100 μm of SOD1 LVPNs (*grey squared*) compared to WT controls (*white circles*) at P65-75 and P120. All data mean ± SEM, with two-way ANOVAs followed by Bonferroni post-tests, *P < 0.05. *n* = 7, 5, 5 and 8 for WT P8-15, P28-35, P65-75 and P120 respectively. *n* = 6, 5, 5 and 12 for SOD1 P8-15, P28-35, P65-75 and P120 respectively. Scale bar: **b**, 50 μm. **c** and **f**, 5 μm

Although the somatosensory cortex did show significant thinning at P120 (Table 1), LVPN dendritic arbors remained unchanged (Fig. 7), showing no differences in the soma volume, total arbor length, apical arbor length, basal arbor length, mean basal tree length, apical reach, basal reach, apical ramifications or basal ramifications at any age studied (Table 5, Fig. 7). However, the dendritic arbors of SOD1 LVPNs within the somatosensory cortex did show significantly decreased apical (Fig. 7c and d) and basal (Fig. 7e and f) spine densities from P65-75 onwards; apical spine density was reduced by 36 % (*P* = 0.031) at P65-75 and by 34 % (*P* = 0.025) at P120 (Table 5, Fig. 7j). Decreased basal dendritic spine density was also present at P120, when there was a 36 % reduction (*P* = 0.042, Table 5).

**Table 5:** Morphometric dendritic and dendritic spine parameters of LVPNs within the somatosensory cortex. All data presented as mean ± SEM

Region	P8-15 (*n*) Neonatal	P28-35 (*n*) Pre-symptomatic	P65-75 (*n*) Onset	P120 (*n*) Mid-disease
Soma volume (μm^3^)	WT: 1339.7 ± 305.4 (*7*)	WT: 1666.5 ± 142.4 (*5*)	WT: 1775.5 ± 54.4 (*5*)	WT: 1666.3 ± 281.3 (*8*)
	SOD1: 1273.1 ± 242.0 (*5*)	SOD1: 2446.7 ± 367.1 (*5*)	SOD1: 24377 ± 96.4 (*5*)	SOD1: 1432.1 ± 192.9 (*12*)
	Not significant	Not significant	Not significant	Not significant
Total arbor length (μm)	WT: 2917 ± 522 (*7*)	WT: 4406 ± 84 (*5*)	WT: 5527 ± 134 (*5*)	WT: 4458 ± 389 (*8*)
	SOD1: 3200 ± 206 (*5*)	SOD1: 5240 ± 492 (*5*)	SOD1: 5818 ± 280 (*5*)	SOD1: 2953 ± 296 (*12*)
	Not significant	Not significant	Not significant	Not significant
Apical arbor length (μm)	WT: 1734 ± 336 (*7*)	WT: 2854 ± 191 (*5*)	WT: 3534 ± 315 (*5*)	WT: 2512 ± 121 (*8*)
	SOD1: 2173 ± 256 (*5*)	SOD1: 2169 ± 143 (*5*)	SOD1: 3828 ± 232 (*5*)	SOD1: 1838 ± 224 (*12*)
	Not significant	Not significant	Not significant	Not significant
Basal arbor length (μm)	WT: 1183 ± 196 (*7*)	WT: 1551 ± 136 (*5*)	WT: 1912 ± 254 (*5*)	WT: 1821 ± 323 (*8*)
	SOD1: 1027 ± 159 (*5*)	SOD1: 2061 ± 400 (*5*)	SOD1: 2331 ± 335 (*5*)	SOD1: 951 ± 90 (*12*)
	Not significant	Not significant	Not significant	Not significant
Mean basal tree length (μm)	WT: 333 ± 56 (*7*)	WT: 469 ± 36 (*5*)	WT: 461 ± 27 (*5*)	WT: 371 ± 53 (*8*)
	SOD1: 326 ± 49 (*5*)	SOD1: 412 ± 43 (*5*)	SOD1: 413 ± 40 (*5*)	SOD1: 253 ± 25 (*12*)
	Not significant	Not significant	Not significant	Not significant
Apical reach (μm)	WT: 564 ± 68 (*7*)	WT: 1213 ± 70 (*5*)	WT: 1265 ± 173 (*5*)	WT: 922 ± 70 (*8*)
	SOD1: 720 ± 21 (*5*)	SOD1: 924 ± 61 (*5*)	SOD1: 1179 ± 197 (*5*)	SOD1: 803 ± 99 (*12*)
	Not significant	Not significant	Not significant	Not significant
Basal reach (μm)	WT: 242 ± 35 (*7*)	WT: 340 ± 36 (*5*)	WT: 293 ± 19 (*5*)	WT: 336 ± 65 (*8*)
	SOD1: 209 ± 18 (*5*)	SOD1: 319 ± 13 (*5*)	SOD1: 299 ± 26 (*5*)	SOD1: 241 ± 24 (*12*)
	Not significant	Not significant	Not significant	Not significant
Apical ramifications	WT: 9.2 ± 0.8 (*7*)	WT: 10 ± 0.6 (*5*)	WT: 14.3 ± 1.8 (*5*)	WT: 10.5 ± 1 (*8*)
	SOD1: 9.4 ± 0.6 (*5*)	SOD1: 12.7 ± 0.8 (*5*)	SOD1: 14.9 ± 1.4 (*5*)	SOD1: 8.4 ± 0.9 (*12*)
	Not significant	Not significant	Not significant	Not significant
Basal ramifications	WT: 4.1 ± 0.3 (*7*)	WT: 4.2 ± 0.5 (*5*)	WT: 4.4 ± 0.3 (*5*)	WT: 4.5 ± 0.5 (*8*)
	SOD1: 4.2 ± 0.5 (*5*)	SOD1: 4.1 ± 0.4 (*5*)	SOD1: 4.5 ± 0.2 (*5*)	SOD1: 3.4 ± 0.2 (*12*)
	Not significant	Not significant	Not significant	Not significant
Apical spine density per 100 μm	WT: 28.9 ± 3.1 (*7*)	WT: 29.4 ± 3.2 (*5*)	WT: 32.8 ± 2.8 (*5*)	WT: 25.7 ± 2.1 (*8*)
	SOD1: 23.1 ± 3.9 (*5*)	SOD1: 19.4 ± 3.3 (*5*)	SOD1: 20.9 ± 2.4 (*5*)	SOD1: 16.9 ± 1.6 (*12*)
	Adj. *P* = 0.57	Adj. *P* = 0.09	Adj. *P* = 0.03*	Adj. *P* = 0.02*
Basal spine density per 100 μm	WT: 21.4 ± 2.1 (*7*)	WT: 22.1 ± 2.2 (*5*)	WT: 27.9 ± 3.3 (*5*)	WT: 20.6 ± 3.1 (*8*)
	SOD1: 16.7 ± 2.9 (*5*)	SOD1: 16.7 ± 3.4 (*5*)	SOD1: 20.3 ± 0.8 (*5*)	SOD1: 13.2 ± 1 (*12*)
	Adj. *P* = 0.79	Adj. *P* = 0.66	Adj. *P* = 0.21	Adj. *P* = 0.04*

All analyses were unpaired two-way ANOVAs for age and genotype of LVPNs from the somatosensory cortex comparing soma volume (Age: *P* = 0.0100*; Genotype: *P* = 0.1295), total dendritic arbor length (Age: *P* < 0.0001****; Genotype: *P* = 0.9331), apical dendritic arbor length (Age: *P* < 0.0001****; Genotype: *P* = 0.6179), basal dendritic arbor length (Age: *P* = 0.0014**; Genotype: *P* = 0.8894), mean basal dendritic tree length (Age: *P* = 0.0048**; Genotype: *P* = 0.0773), apical dendritic reach (Age: *P* < 0.0001****; Genotype: *P* = 0.2972), basal dendritic reach (Age: *P* = 0.1037; Genotype: *P* = 0.2159), apical branch ramifications (Age: *P* = 0.0011**; Genotype: *P* = 0.7403), basal branch ramifications (Age: *P* = 0.7061; Genotype: *P* = 0.4196), apical dendritic spine density per 100 μm (Age: *P* = 0.1270; Genotype: *P* < 0.0001****) and basal dendritic spine density per 100 μm (Age: *P* = 0.0343*; Genotype: *P* = 0.0009***). Bonferroni post-tests were used to compare the effect of genotype at each age group, with the adjusted *P*-value (adj. *P*) reported in the table for parameters that had significant genotype effects. Each animal used (*n*) contains mean values from a minimum of 2 individual neurons, specifically P8-15 (WT: 15 neurons; SOD1: 10 neurons), P28-35 (WT: 12 neurons; SOD1: 17 neurons), P65-75 (WT: 14 neurons; SOD1: 12 neurons) and P120 (WT: 20 neurons; SOD1: 28 neurons)

Taken together, these data show a loss of dendritic spines in LVPNs of the somatosensory cortex in SOD1 mice, in the absence of any changes in dendritic structure. As our observations in the motor cortex and MPFC consistently show reduction in spine density preceding, or concurrent with, dendritic degeneration (see Tables 2, 3 and 4), spine density reduction may reflect an underlying synaptic mechanism common to motor, cognitive and sensory dysfunction in ALS.

### Somatosensory cortex LII-IIIPNs in SOD1 mice show no changes in their dendrites or spine density at any age studied

By contrast to the changes we observed in other pyramidal neurons in motor cortex, the MPFC and somatosensory cortex, there were no differences in the soma volume, total arbor length, apical arbor length, basal arbor length, mean basal tree length, apical reach, basal reach, apical ramifications, basal ramifications, apical spine density or basal spine density of somatosensory cortex LII-IIIPNs in SOD1 mice at any age studied (Table 6). This finding shows that changes in other pyramidal neurons are highly likely to be related to disease-specific factors. To provide further evidence to support this, we next investigated whether pyramidal neurons from the entorhinal cortex, a region that is functionally related to memory processing and storage and which is frequently dysfunctional in dementia [67].

**Table 6:** Morphometric dendritic and dendritic spine parameters of LII-IIIPNs within the somatosensory cortex. All data presented as mean ± SEM

Region	P8-15 (*n*) Neonatal	P28-35 (*n*) Pre-symptomatic	P65-75 (*n*) Onset	P120 (*n*) Mid-disease
Soma volume (μm^3^)	WT: 749.7 ± 139.9 (*7*)	WT: 1051.6 ± 149.9 (*5*)	WT: 1119.7 ± 54.4 (*5*)	WT: 929.9 ± 281.3 (*8*)
	SOD1: 954.5 ± 261.2 (*5*)	SOD1: 1127.8 ± 304.3 (*5*)	SOD1: 823.8 ± 96.4 (*5*)	SOD1: 853.8 ± 192.9 (*12*)
	Not significant	Not significant	Not significant	Not significant
Total arbor length (μm)	WT: 2355 ± 366 (*7*)	WT: 2408 ± 324 (*5*)	WT: 2410 ± 323 (*5*)	WT: 2869 ± 311 (*8*)
	SOD1: 2044 ± 416 (*5*)	SOD1: 2405 ± 402 (*5*)	SOD1: 2466 ± 483 (*5*)	SOD1: 2176 ± 187 (*12*)
	Not significant	Not significant	Not significant	Not significant
Apical arbor length (μm)	WT: 1081 ± 115 (*7*)	WT: 1172 ± 187 (*5*)	WT: 1186 ± 172 (*5*)	WT: 1654 ± 207 (*8*)
	SOD1: 1020 ± 162 (*5*)	SOD1: 949 ± 103 (*5*)	SOD1: 1000 ± 231 (*5*)	SOD1: 1225 ± 96 (*12*)
	Not significant	Not significant	Not significant	Not significant
Basal arbor length (μm)	WT: 1317 ± 236 (*7*)	WT: 1236 ± 149 (*5*)	WT: 1224 ± 154 (*5*)	WT: 1159 ± 124 (*8*)
	SOD1: 968 ± 277 (*5*)	SOD1: 1455 ± 369 (*5*)	SOD1: 1466 ± 267 (*5*)	SOD1: 951 ± 119 (*12*)
	Not significant	Not significant	Not significant	Not significant
Mean basal tree length (μm)	WT: 389 ± 47 (*7*)	WT: 335 ± 48 (*5*)	WT: 313 ± 32 (*5*)	WT: 325 ± 38 (*8*)
	SOD1: 293 ± 102 (*5*)	SOD1: 364 ± 95 (*5*)	SOD1: 341 ± 38 (*5*)	SOD1: 235 ± 29 (*12*)
	Not significant	Not significant	Not significant	Not significant
Apical reach (μm)	WT: 323 ± 23 (*7*)	WT: 486 ± 65 (*5*)	WT: 501 ± 66 (*5*)	WT: 528 ± 56 (*8*)
	SOD1: 463 ± 67 (*5*)	SOD1: 367 ± 12 (*5*)	SOD1: 455 ± 61 (*5*)	SOD1: 468 ± 57 (*12*)
	Not significant	Not significant	Not significant	Not significant
Basal reach (μm)	WT: 202 ± 18 (*7*)	WT: 234 ± 30 (*5*)	WT: 236 ± 22 (*5*)	WT: 207 ± 13 (*8*)
	SOD1: 205 ± 51 (*5*)	SOD1: 264 ± 48 (*5*)	SOD1: 239 ± 35 (*5*)	SOD1: 196 ± 22 (*12*)
	Not significant	Not significant	Not significant	Not significant
Apical ramifications	WT: 6.8 ± 1.2 (*7*)	WT: 5.7 ± 0.7 (*5*)	WT: 7.2 ± 0.5 (*5*)	WT: 9.2 ± 1 (*8*)
	SOD1: 8 ± 0.7 (*5*)	SOD1: 5.3 ± 0.5 (*5*)	SOD1: 5.5 ± 1.2 (*5*)	SOD1: 5.2 ± 0.5 (*12*)
	Not significant	Not significant	Not significant	Not significant
Basal ramifications	WT: 4.3 ± 0.4 (*7*)	WT: 3.8 ± 0.4 (*5*)	WT: 4.1 ± 0.1 (*5*)	WT: 4.3 ± 0.3 (*8*)
	SOD1: 3.7 ± 0.4 (*5*)	SOD1: 5.1 ± 1 (*5*)	SOD1: 4.3 ± 0.4 (*5*)	SOD1: 3.7 ± 0.2 (*12*)
	Not significant	Not significant	Not significant	Not significant
Apical spine density per 100 μm	WT: 30.5 ± 1.4 (*7*)	WT: 31.6 ± 3.1 (*5*)	WT: 34 ± 2.1 (*5*)	WT: 25.8 ± 3.4 (*8*)
	SOD1: 25.5 ± 3.1 (*5*)	SOD1: 32.8 ± 4.4 (*5*)	SOD1: 28.2 ± 6.6 (*5*)	SOD1: 24.6 ± 2.5 (*12*)
	Not significant	Not significant	Not significant	Not significant
Basal spine density per 100 μm	WT: 21.6 ± 2.5 (*7*)	WT: 29.6 ± 6.9 (*5*)	WT: 24.2 ± 1.7 (*5*)	WT: 17.4 ± 2.1 (*8*)
	SOD1: 23.6 ± 4.7 (*5*)	SOD1: 22.2 ± 3.1 (*5*)	SOD1: 18.8 ± 4 (*5*)	SOD1: 16.4 ± 1.5 (*12*)
	Not significant	Not significant	Not significant	Not significant

All analyses were unpaired two-way ANOVAs for age and genotype of LII-IIIPNs from the somatosensory cortex comparing soma volume (Age: *P* = 0.5903; Genotype: *P* = 0.8586), total dendritic arbor length (Age: *P* = 0.7853; Genotype: *P* = 0.3398), apical dendritic arbor length (Age: *P* = 0.0271*; Genotype: *P* = 0.0643), basal dendritic arbor length (Age: *P* = 0.3671; Genotype: *P* = 0.8741), mean basal dendritic tree length (Age: *P* = 0.5589; Genotype: *P* = 0.6085), apical dendritic reach (Age: *P* = 0.2468; Genotype: *P* = 0.6187), basal dendritic reach (Age: *P* = 0.2883; Genotype: *P* = 0.7704), apical branch ramifications (Age: *P* = 0.0493*; Genotype: *P* = 0.1262), basal branch ramifications (Age: *P* = 0.7336; Genotype: *P* = 0.8754), apical dendritic spine density per 100 μm (Age: *P* = 0.1546; Genotype: *P* = 0.2195) and basal dendritic spine density per 100 μm (Age: *P* = 0.0328*; Genotype: *P* = 0.2041). Bonferroni post-tests were used to compare the effect of genotype at each age group, with the adjusted *P*-value (adj. *P*) reported in the table for parameters that had significant genotype effects. Each animal used (*n*) contains mean values from multiple individual neurons, specifically P8-15 (WT: 19 neurons; SOD1: 8 neurons), P28-35 (WT: 9 neurons; SOD1: 10 neurons), P65-75 (WT: 10 neurons; SOD1: 11 neurons) and P120 (WT: 16 neurons; SOD1: 24 neurons)

### Entorhinal cortex LII-IIIPNs in SOD1 mice show no changes in dendritic structure or spine density

There were no differences in the soma volume, total arbor length, apical arbor length, basal arbor length, mean basal tree length, apical reach, basal reach, apical ramifications, basal ramifications, apical spine density or basal spine density of entorhinal cortex LII-IIIPNs in SOD1 mice compared to WT controls at any age studied (Table 7). Thus, as for LII-IIIPNs from the somatosensory cortex, LII-IIIPNs in entorhinal cortex are spared from any disease-related pathology at all ages studied, further supporting the hypothesis that changes in other cortical pyramidal neurons are driven by disease-specific processes.

**Table 7:** Morphometric dendritic and dendritic spine parameters of LII-IIIPNs within the entorhinal cortex. All data presented as mean ± SEM

Region	P8-15 (*n*) Neonatal	P28-35 (*n*) Pre-symptomatic	P65-75 (*n*) Onset	P120 (*n*) Mid-disease
Soma volume (μm^3^)	WT: 1172.4 ± 2198 (*7*)	WT: 1328.1 ± 1498 (*5*)	WT: 1162.2 ± 714 (*5*)	WT: 1132.7 ± 332.7 (*8*)
	SOD1: 840.7 ± 1441 (*5*)	SOD1: 1045.4 ± 873 (*5*)	SOD1: 1232.2 ± 4599 (*5*)	SOD1: 1047.1 ± 409.5 (*12*)
	Not significant	Not significant	Not significant	Not significant
Total arbor length (μm)	WT: 2691 ± 244 (*7*)	WT: 3166 ± 218 (*5*)	WT: 3732 ± 543 (*5*)	WT: 3150 ± 109 (*8*)
	SOD1: 2229 ± 414 (*5*)	SOD1: 3375 ± 309 (*5*)	SOD1: 3145 ± 597 (*5*)	SOD1: 3235 ± 171 (*12*)
	Not significant	Not significant	Not significant	Not significant
Apical arbor length (μm)	WT: 1197 ± 203 (*7*)	WT: 1754 ± 122 (*5*)	WT: 1881 ± 277 (*5*)	WT: 1651 ± 99 (*8*)
	SOD1: 1123 ± 271 (*5*)	SOD1: 1515 ± 205 (*5*)	SOD1: 1481 ± 212 (*5*)	SOD1: 1636 ± 99 (*12*)
	Not significant	Not significant	Not significant	Not significant
Basal arbor length (μm)	WT: 1493 ± 148 (*7*)	WT: 1394 ± 161 (*5*)	WT: 1831 ± 314 (*5*)	WT: 1506 ± 126 (*8*)
	SOD1: 1105 ± 170 (*5*)	SOD1: 1859 ± 281 (*5*)	SOD1: 1657 ± 696 (*5*)	SOD1: 1548 ± 77 (*12*)
	Not significant	Not significant	Not significant	Not significant
Mean basal tree length (μm)	WT: 501 ± 69 (*7*)	WT: 342 ± 14 (*5*)	WT: 400 ± 71 (*5*)	WT: 351 ± 24 (*8*)
	SOD1: 408 ± 103 (*5*)	SOD1: 515 ± 51 (*5*)	SOD1: 348 ± 135 (*5*)	SOD1: 413 ± 24 (*12*)
	Not significant	Not significant	Not significant	Not significant
Apical reach (μm)	WT: 543 ± 36 (*7*)	WT: 772 ± 74 (*5*)	WT: 619 ± 63 (*5*)	WT: 565 ± 13 (*8*)
	SOD1: 520 ± 70 (*5*)	SOD1: 590 ± 45 (*5*)	SOD1: 760 ± 54 (*5*)	SOD1: 409 ± 38 (*12*)
	Not significant	Not significant	Not significant	Not significant
Basal reach (μm)	WT: 166 ± 16 (*7*)	WT: 131 ± 6 (*5*)	WT: 173 ± 22 (*5*)	WT: 137 ± 9 (*8*)
	SOD1: 136 ± 26 (*5*)	SOD1: 193 ± 11 (*5*)	SOD1: 126 ± 24 (*5*)	SOD1: 152 ± 17 (*12*)
	Not significant	Not significant	Not significant	Not significant
Apical ramifications	WT: 5.3 ± 0.6 (*7*)	WT: 7.1 ± 0.6 (*5*)	WT: 7.4 ± 0.2 (*5*)	WT: 8.3 ± 1.6 (*8*)
	SOD1: 6 ± 0.6 (*5*)	SOD1: 5.8 ± 0.8 (*5*)	SOD1: 8.9 ± 0.9 (*5*)	SOD1: 7.3 ± 0.2 (*12*)
	Not significant	Not significant	Not significant	Not significant
Basal ramifications	WT: 6 ± 1.1 (*7*)	WT: 4.8 ± 0.3 (*5*)	WT: 4 ± 0.8 (*5*)	WT: 5 ± 0.3 (*8*)
	SOD1: 5.8 ± 1.3 (*5*)	SOD1: 5 ± 0.5 (*5*)	SOD1: 3.5 ± 0.7 (*5*)	SOD1: 4.7 ± 0.2 (*12*)
	Not significant	Not significant	Not significant	Not significant
Apical spine density per 100 μm	WT: 52.3 ± 7.2 (*7*)	WT: 39.8 ± 1.7 (*5*)	WT: 23.2 ± 2.2 (*5*)	WT: 28 ± 2.1 (*8*)
	SOD1: 51.8 ± 8.8 (*5*)	SOD1: 39.6 ± 4.4 (*5*)	SOD1: 33.6 ± 4.9 (*5*)	SOD1: 35.7 ± 3.5 (*12*)
	Not significant	Not significant	Not significant	Not significant
Basal spine density per 100 μm	WT: 55.6 ± 6 (*7*)	WT: 41.2 ± 3.7 (*5*)	WT: 23.4 ± 2.4 (*5*)	WT: 35.8 ± 3 (*8*)
	SOD1: 60.6 ± 9.7 (*5*)	SOD1: 40 ± 3.4 (*5*)	SOD1: 35 ± 5.1 (*5*)	SOD1: 39.3 ± 2.9 (*12*)
	Not significant	Not significant	Not significant	Not significant

All analyses were unpaired two-way ANOVAs for age and genotype of LII-IIIPNs from the entorhinal cortex comparing soma volume (Age: *P* = 0.0010**; Genotype: *P* = 0.4552), total dendritic arbor length (Age: *P* = 0.0301*; Genotype: *P* = 0.4354), apical dendritic arbor length (Age: *P* = 0.0256*; Genotype: *P* = 0.9445), basal dendritic arbor length (Age: *P* = 0.0301*; Genotype: *P* = 0.4354), mean basal dendritic tree length (Age: *P* = 0.5982; Genotype: *P* = 0.6433), apical dendritic reach (Age: *P* = 0.0002***; Genotype: *P* = 0.1269), basal dendritic reach (Age: *P* = 0.7915; Genotype: *P* = 0.9994), apical branch ramifications (Age: *P* = 0.0139*; Genotype: *P* = 0.9351), basal branch ramifications (Age: *P* = 0.0742; Genotype: *P* = 0.7342), apical dendritic spine density per 100 μm (Age: *P* = 0.0001****; Genotype: *P* = 0.1906) and basal dendritic spine density per 100 μm (Age: *P* = 0.0002***; Genotype: *P* = 0.2384). Bonferroni post-tests were used to compare the effect of genotype at each age group, with the adjusted *P*-value (adj. *P*) reported in the table for parameters that had significant genotype effects. Each animal used (*n*) contains mean values from multiple individual neurons, specifically P8-15 (WT: 11 neurons; SOD1: 9 neurons), P28-35 (WT: 11 neurons; SOD1: 7 neurons), P65-75 (WT: 6 neurons; SOD1: 7 neurons) and P120 (WT: 13 neurons; SOD1: 14 neurons)

## Discussion

This study is the first longitudinal investigation of changes in the dendritic arborisation and spine density of individual cortical pyramidal neurons in vulnerable and non-vulnerable populations in any ALS rodent model. We report several novel findings. While motor cortex LVPNs, which will include canonical upper MNs but also cells with other output targets, showed the most extensive morphological changes, LII-IIIPNs in motor cortex, LVPNs in somatosensory cortex and MPFC pyramidal neurons all showed significant early alterations in SOD1 mice. While most pyramidal neuron populations showed only progressive dendritic shrinkage or spine loss, neonatal MPFC pyramidal neurons first exhibited a selective increase in the length of their basal dendritic arbour, before undergoing dendritic shrinkage and spine loss. By contrast, LII-IIIPNs in somatosensory or entorhinal cortex were unaltered in SOD1 mice, underlining the specificity of morphological alterations in other cortical areas. Our demonstration of morphological changes in the dendritic arbors of neurons within the motor cortex, MPFC, and somatosensory cortex support the hypothesis that activity-dependent mechanisms are a significant driver of both motor and non-motor phenotypes in ALS, and broadly support glutamate-induced excitotoxic processes as a primary pathology of ALS.

### The most extensive changes occur in motor cortex pyramidal neurons

Recent human neuroimaging studies have reported extensive cortical thinning in frontal lobe gyri, including the precentral gyrus [50, 55, 56, 62, 78]. While the lack of cortical gyrification in mice makes direct comparison to humans difficult [70], we found that cortical thinning appeared earliest in the primary motor cortex of SOD1 mice, occurring at P28-35. Motor cortex thinning was largely due to LVPN apical dendrite changes, as thinning presented simultaneously with decreased total arbor length, apical arbor length and apical spine density in motor cortex LVPNs; thinning persisted at all stages, including the oldest age studied, P120, but did not increase as later morphological changes, including decreased apical dendritic reach, mean basal tree lengths and basal dendrite spine densities, became apparent in SOD1 LVPNs.

The apical dendrites of motor cortex LVPNs predominantly receive excitatory inputs from the thalamus and other cortical areas, while the basal dendrites receive excitatory inputs from LII/IIIPNs and other LVPNs [75]. Our observation of early decreases in apical dendrite length and apical spine density suggests that thalamic and intra-cortical inputs will be preferentially altered in pre-symptomatic SOD1 mice, while the onset of decreased basal dendrite spine density and tree length at symptom onset suggests that inputs from layer II/III and layer V will only be altered at later stages.

Motor cortex LII-IIIPNs showed the earliest onset of apical spine density reduction, compared to WT controls, at P8-15; this reduction increased at P28-35 and P65-75, but age-related decline in apical spine density at P120 in wild type mice meant that there was no significant difference in SOD1 mice at this age. LII/IIIPNs also showed reductions in total arbor length and apical arbor length at P120, suggesting that thalamic and intra-cortical inputs will be altered later in disease progression.

Significant corticospinal neuron loss occurs in SOD1 mice from P60 onwards [45, 76], with dendritic spine loss commencing from P21-27 [20] and apical dendritic degeneration from P28-40 [20]. It is important to note that significant structural alterations in identified UMNs occur prior to P60, with decreased somatic cross sectional diameter from P30 [45] and lower (although not significant) UMN numbers, compared to controls occur at P30 in this rodent model [45]. We did not observe somatic volume alteration at P30, most likely due to differences in methodology; our Golgi method was optimised for dendritic impregnation, so somas were over-stained and somatic morphometry compromised, and we used somatic volume estimates, compared to single diameter measurements. The variability in our measurements may also be influenced by the fact that our neuronal samples include populations of intra cortical and other non-spinal projection neurons that are resistant to degeneration [45]. The same study also reported no loss of cortical interneurons or layer II-III pyramidal neurons [45].

In other morphological studies at single time points in disease progression, basal dendritic lengthening at P26-31 [53], apical dendritic regression and spine loss at P65 [30], and basal dendritic arbor shortening at P80-85 [58] have been reported. Taking differences in methodology into consideration, our quantifications of dendrite length are in good agreement with previous morphological descriptions of motor cortex LVPNs in SOD1 and wild type mice [20, 30], although our spine density estimates are somewhat lower (in both genotypes) than those generated using high-resolution spinning disk confocal microscopy [20]. In interpreting alterations in dendritic length and spine density estimates, some caution must be taken when applying the terms ‘*dendritic loss*’ or ‘*spine loss*’, as our measurement time points encompass a developmental period of rapid change. In particular, the dendrites and dendritic spine density of wild type cortical pyramidal neurons generally increase rapidly from birth to P14, and then more slowly to P70, followed by a decline [49, 51]. By contrast, while pyramidal neurons in neonatal SOD1 mice overall initially showed similar dendrite length and spine density at P8-15, further increases in vulnerable pyramidal neuron populations were either reduced or delayed. It is thus possible that reductions in dendritic arbor and spine density are a developmental deficit, rather than loss of existing structures. Support for pathogenic spine loss in the SOD1 mouse, as opposed to developmental deficits, include the lack of structural difference in dendrites and spines in SOD1 motor cortex LVPNs at P8-15, and the observation that, in less vulnerable neuronal pools, lowered dendritic length or spine density occurs much later, if at all (see results for LII-II motor cortex, somatosensory cortex and entorhinal cortex). In addition, electrophysiological changes in different strains of SOD1 mice are already present during immediate neonatal periods [4, 40, 46, 69] and throughout postnatal development [20, 69], potentially altering dendritic and dendritic spine loss due to activity-dependent postnatal developmental pruning [5, 9, 26, 28, 54]. Consistent with our current understanding of synaptic plasticity [26, 54, 59], increased excitatory neurotransmission during development should lead to increased dendrites and/or dendritic spine density before spine loss occurs, such as in late embryonic early postnatal lower MNs [19], although, in SOD1 strains, developmental increases or decreases in dendritic arbor length compared to controls remains controversial [3, 4, 17, 36, 40]. Hyperexcitability (by either reduced inhibition or increased excitation) is also prevalent in non-symptomatic human carriers of ALS-associated mutations [22, 73].

Although we have not determined whether the motor cortex LVPNs sampled have corticospinal projections as shown by retrograde labelling [30, 45], more than 45 % of the motor cortex LVPN population is retrogradely labelled by corticospinal tract injection [61]. Our observations in SOD1 mice are therefore consistent with a selective vulnerability of corticospinal neurons in ALS, as seen in human pathology [24, 27, 65]. It is worth noting that human Golgi studies of Betz cell loss [24] in ALS are similarly unable to distinguish between corticospinal neurons and other large LVPNs with different target projections. The morphological changes seen in LVPNs are consistent with those of glutamate-induced excitotoxicity [8]. LII/IIIPNs in the motor cortex were also altered, showing the earliest decrease in apical spine density of all cortical neurons examined, as well as apical and total arbor length reduction later in disease progress. As LII/IIIPNs project to both ipsi- and contra-lateral upper MNs, neuro-motor function will be further compromised by these changes [9, 30].

### The MPFC

Imaging studies of ALS patients have shown cortical thinning in prefrontal cortex areas [56, 62, 78], consistent with deficits in executive function [1, 6]. SOD1 mice also showed cortical thinning by mid-disease, suggesting that tests of executive function, such as spatial memory or working memory [66], may also be impaired. Pyramidal neurons in the MPFC of SOD1 mice showed a unique morphological signature, with a selective neonatal increase in basal dendrite total length and tree length, followed by progressive regression of basal dendrites, so that by symptom onset, total arbor length, total basal dendrite length and mean basal tree length were reduced; apical reach was also reduced, but not until mid-disease, consistent with the onset of cortical thinning. Decreased dendrite spine density was first seen in basal dendrites at pre-symptomatic ages, followed by apical dendrites at symptom onset.

Taken together, these results indicate a later onset of regressive changes in MPFC pyramidal neurons, compared to motor cortex LVPNs, with basal dendrites affected first in the former, while apical dendrites were affected first in the latter. Intriguingly, MPFC pyramidal neurons were the only cortical neurons that showed an increase in dendrite length at any age, perhaps reflecting compensatory or, alternatively, maladaptive alterations in this cortical regions.

Selective reduction in basal dendrite arbors and dendritic spine loss have been previously reported at P85-90 in SOD1 mice, where these morphological changes were behaviourally correlated with reduced ability to extinguish fear conditioning [57]. Although the method of arbor analysis differed, our apical reach and basal reach measurements are in good agreement with the distal Scholl radius extent reported, and we find the same trend in spine density, albeit with generally lower values due to differences in tracing magnifications [57].

Our findings thus show that changes in the MPFC of SOD1 mice correlate with cortical thinning and executive function deficits seen in some ALS patients [18, 37, 38, 47], strengthening the correspondence between the phenotype of this model and that of human ALS.

### The somatosensory and entorhinal cortex

Somatosensory processing is commonly held to be relatively intact in ALS, despite reports of pyramidal neuron loss and cortical thinning in primary somatosensory areas [2, 42, 62]. We also found significant cortical thinning in the somatosensory cortex of SOD1 mice by mid disease, although there were no significant concurrent dendritic arbor changes at any age studied. Although somatosensory cortex LVPN dendrite lengths were unchanged in SOD1 mice, spine density was significantly decreased, first in apical dendrites at symptom onset, and then in basal dendrites at P120.

While we measured dendrite length and spine density alterations in LII-IIIPNs of the somatosensory and entorhinal cortex, we did not find any significant differences between wild type and SOD1 mice. This result reinforces the specificity of the other morphological changes we have described, and shows that specific populations of cortical pyramidal neurons are vulnerable in SOD1 mice. Although, to date, no assessment of somatosensory pyramidal neuron numbers in rodent models of ALS has been published, the hippocampus, which has major connective pathways to the entorhinal cortex has been shown to have loss of interneurons by P56 [48].

## Conclusions

In summary, we report the first longitudinal study of cortical thinning and of changes in the dendritic arbors and dendritic spines of pyramidal neurons in multiple areas of cortex in the SOD1 mouse model of ALS. Significant morphological changes began at neonatal or pre-symptomatic ages (P8-15 or P28-35) in LVPNs and LII-IIIPNs of the motor cortex and pyramidal neurons of the MPFC. Spine loss without concurrent dendritic regression was also present in somatosensory LVPNs at disease-onset (P65-75).

Characterising the onset, progression and nature of alterations in dendritic arbors and dendritic spine alterations has allowed us to define changes in the motor cortex of SOD1 mice, strengthening the correspondence between the phenotype of this mouse model and that of human ALS. In addition, we have also identified other cortical areas, outside of the motor cortex, where significant effects are likely to occur in ALS, and where electrophysiological or neurobehavioral investigation may prove fruitful. Indeed, validated stereological assessments of neuronal numbers in all brain regions implicated in the ALS – fronto-temporal dementia continuum would be useful both post-mortem and in newer animal models of ALS. The morphological changes seen here are consistent with increases in glutamatergic excitatory neurotransmission previously shown at early ages in this model [20, 53, 69] and with human induced pluripotent stem cell studies of ALS patients [14]. We hope this study will serve as a useful reference point for further characterisation of cellular and functional changes in other ALS and fronto-temporal dementia models, in addition to being a necessary and powerful baseline to gauge the functional and behavioural effects of potential therapeutics.
